# Identifying disulfidptosis subtypes in hepatocellular carcinoma through machine learning and preliminary exploration of its connection with immunotherapy

**DOI:** 10.1186/s12935-024-03387-1

**Published:** 2024-06-03

**Authors:** Guanjun Chen, Ganghua Zhang, Yuxing Zhu, Anshan Wu, Jianing Fang, Zhijing Yin, Haotian Chen, Ke Cao

**Affiliations:** 1 Department of Oncology, Third Xiangya Hospital of Central South University, Changsha, 410013 China; 2 Department of Oncology,, Zhuzhou Hospital Xiangya School of Medicine, Zhuzhou, 412000 China

**Keywords:** Hepatocellular carcinoma, Disulfidptosis, Molecular typing, Survival prognosis, Immunotherapy

## Abstract

**Background:**

Hepatocellular carcinoma (HCC) is a highly prevalent and deadly cancer, with limited treatment options for advanced-stage patients. Disulfidptosis is a recently identified mechanism of programmed cell death that occurs in SLC7A11 high-expressing cells due to glucose starvation-induced disintegration of the cellular disulfide skeleton. We aimed to explore the potential of disulfidptosis, as a prognostic and therapeutic marker in HCC.

**Methods:**

We classified HCC patients into two disulfidptosis subtypes (C1 and C2) based on the transcriptional profiles of 31 disulfrgs using a non-negative matrix factorization (NMF) algorithm. Further, five genes (*NEIL3, MMP1, STC2, ADH4* and *CFHR3*) were screened by Cox regression analysis and machine learning algorithm to construct a disulfidptosis scoring system (disulfS). Cell proliferation assay, F-actin staining and PBMC co-culture model were used to validate that disulfidptosis occurs in HCC and correlates with immunotherapy response.

**Results:**

Our results suggests that the low disulfidptosis subtype (C2) demonstrated better overall survival (OS) and progression-free survival (PFS) prognosis, along with lower levels of immunosuppressive cell infiltration and activation of the glycine/serine/threonine metabolic pathway. Additionally, the low disulfidptosis group showed better responses to immunotherapy and potential antagonism with sorafenib treatment. As a total survival risk factor, disulfS demonstrated high predictive efficacy in multiple validation cohorts. We demonstrated the presence of disulfidptosis in HCC cells and its possible relevance to immunotherapeutic sensitization.

**Conclusion:**

The present study indicates that novel biomarkers related to disulfidptosis may serve as useful clinical diagnostic indicators for liver cancer, enabling the prediction of prognosis and identification of potential treatment targets.

**Graphical Abstract:**

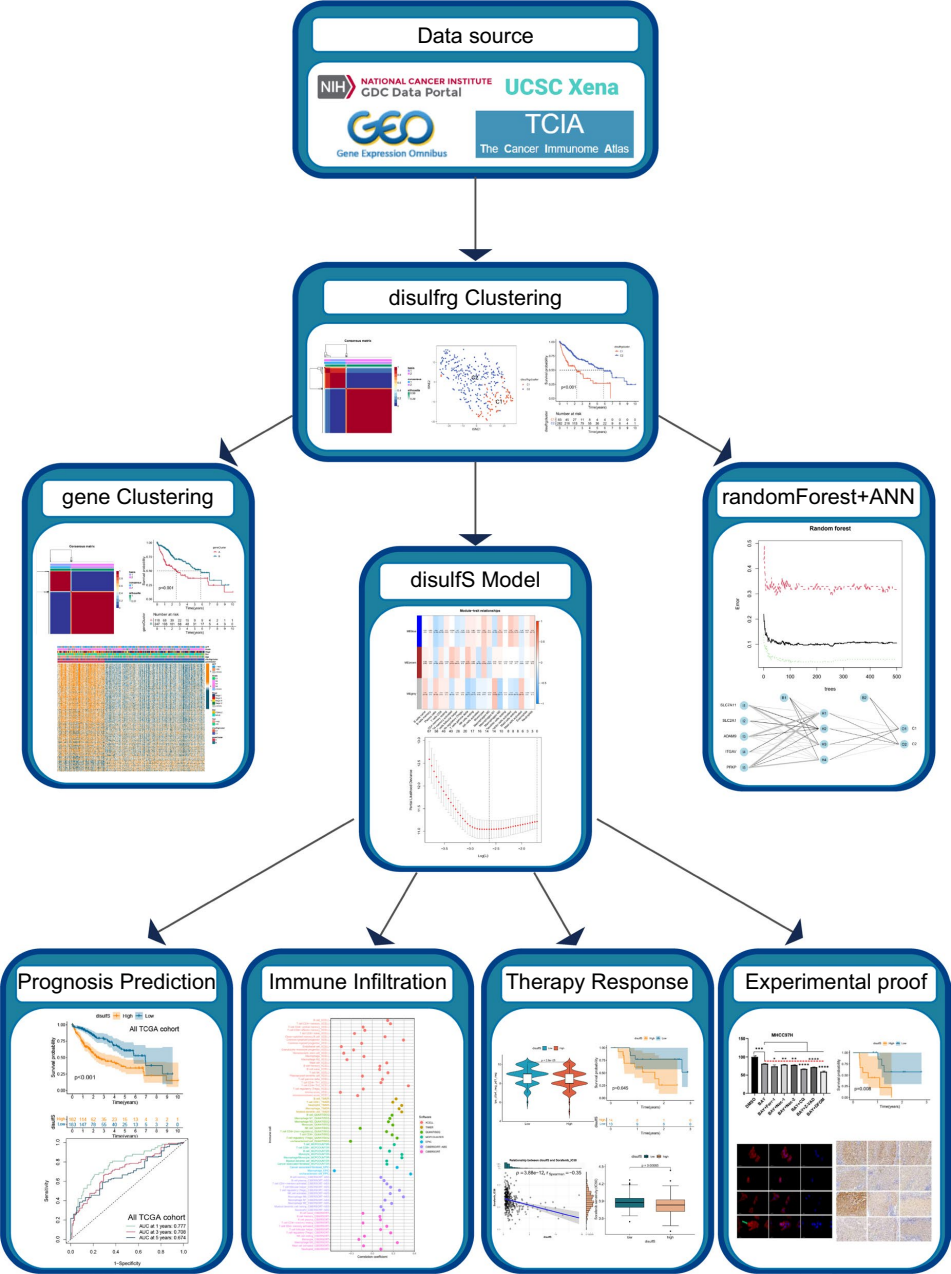

**Supplementary Information:**

The online version contains supplementary material available at 10.1186/s12935-024-03387-1.

## Introduction

Liver cancer, characterized by a high mortality rate, is among the most prevalent gastrointestinal malignancies worldwide. According to a global epidemiological study on tumors, hepatocellular carcinoma (HCC), the primary type of liver cancer, ranks fifth in terms of incidence and third in terms of mortality [1]. The poor prognosis of HCC is attributed to its significant heterogeneity, propensity for metastasis, and overall physical deterioration [2]. While chronic hepatitis B virus infection is the primary cause of HCC development in Asia, chronic hepatitis C virus, alcoholic hepatic steatosis, and nonalcoholic steatohepatitis are the primary causes in Western countries. Unfortunately, patients often seek medical attention only when they exhibit significant symptoms, by which time the disease has usually reached advanced stages [3, 4]. Despite the development of various therapeutic modalities for HCC treatment over the past decades, the five-year survival rate remains dismally low at 14.1% [5, 6].

The tumor microenvironment (TME) in HCC is complex, and the interplay between multiple immune cells and stromal cells creates an immunosuppressive microenvironment, leading to unfavorable outcomes in immunotherapy for HCC [7]. In recent years, immunotherapy based on immune checkpoint inhibitors (ICIs) has emerged as a highly promising approach for treating various cancers. ICIs restore the activity of immune cells, effectively eliminating tumors [7]. Several studies have demonstrated the clinical efficacy of ICIs in advanced HCC [8, 9]. However, due to the genetic, metabolic, and immune heterogeneity of HCC, conventional molecular typing has limitations in identifying populations that would benefit from ICI therapy. Hence, understanding the genomic profile of HCC and developing more effective and reliable prognostic markers are crucial for enhancing current treatment strategies and prolonging patient survival.

Tumor cells undergo metabolic reprogramming, rendering them highly dependent on specific nutrients such as glucose and glutamine [10]. The main glucose metabolic pathways in tumor cells include glycolysis, the pentose phosphate pathway, and the tricarboxylic acid cycle. Through these pathways, tumor cells generate ATP for growth and development and obtain precursor molecules for biomolecule synthesis. Upregulation of glucose transporter protein expression, especially *SLC2A1* and *SLC2A3*, is a hallmark of tumor metabolic reprogramming [11]. Most cancer cells uptake extracellular cystine via the cystine transporter protein system Xc- (composed of the catalytic subunit *SLC7A11* and the chaperone subunit *SLC3A2*) and subsequently utilize intracellular NADPH to reduce cystine to cysteine for cellular utilization [12]. However, in cancer cells with high *SLC7A11* expression, the low solubility and potential toxicity of intracellular cystine lead to accelerated reduction to the more soluble cysteine, consuming significant amounts of intracellular NADPH [13]. Under glucose starvation conditions, high *SLC7A11*-expressing cancer cells experience depletion of the intracellular NADPH pool, resulting in the accumulation of intracellular cystine and other disulfides, leading to rapid cell death [14]. Recent research elucidated the detailed mechanism of this type of cell death, which is referred to as disulfidptosis. In this mode of cell death, inhibitors of iron death, apoptosis, necrosis, and autophagy pathways failed to rescue glucose starvation-induced tumor cell death in *SLC7A11* overexpressing cells. It was found that NADPH depletion caused aberrant disulfide bonding in actin cytoskeletal proteins, leading to actin network collapse and subsequent cell death [15]. In HCC, the study of disulfidptosis, a regulated form of cell death, remains limited. Only a few studies have utilized machine learning techniques to construct tumor prognostic signatures based on disulfidptosis-related genes (disulfrg) for clinical treatment guidance and risk stratification [16–19]. Consequently, there is an urgent need to develop a novel prognostic signature for disulfidptosis in HCC and validate it through molecular biology experiments and analysis of clinical cohorts. Such efforts aim to identify a practical biomarker that can effectively guide treatment strategies for patients with HCC in a clinical setting.

In this study, we conducted a series of investigations based on disulfrg and employed multiple algorithms to construct a novel HCC prognostic marker called the disulfS. This score effectively reflects the disulfidptosis status and survival of patients. We comprehensively validated the score and found it reliable in predicting clinical prognosis, microsatellite instability (MSI), tumor stemness index, immune infiltration, and immunotherapy response. Moreover, we investigated the occurrence of disulfidptosis in HCC cells through rigorous molecular biology experiments. To validate the effectiveness of disulfS, clinical samples from our institution were utilized for comprehensive analysis and evaluation. Our study successfully developed a novel disulfS, enabling effective staging of disulfidptosis in HCC patients and aiding in the planning of immunotherapy regimens and patient management for improved individualized treatment.

## Materials and methods

### Data collection and cleaning

We collected 31 disulfrgs from a previous article [15], including *SLC7A11, SLC3A2, RPN1, NCKAP1, RAC1, WASF-2, CYFIP1, ABI2, BRK1, SLC2A1, GYS1, OXSM, NDUFS1, NDUFA11, NUBPL, LRPPRC, PRDX1, FLNA, FLNB, MYH9, MYH10, TLN1, ACTB, CD2AP, INF2, ACTN4, PDLIM1, IQGAP1, DSTN, CAPZB,* and *MYL6*. We obtained the “TCGA-LIHC” liver cancer cohort data from the GDC database (https://portal.gdc.cancer.gov/), containing expression profiles of 371 tumor samples and 50 normal samples. We selected 365 liver cancer samples with complete expression profiles and clinical survival data and randomly divided them into training and internal validation sets in a 6:4 ratio. We converted the expression profile data from FPKM to TPM and collated it into log2 (TPM + 1) format. We used 216 samples from GSE15654 as the external validation set and 27 samples from the GSE78220 melanoma PD-1 inhibitor treatment cohort for immunotherapy efficacy validation. Immunophenoscore (IPS) for ICIs were obtained from the TCIA database (https://tcia.at). We obtained simple nucleotide variation data in mutation annotation maf format from the GDC database and calculated TMB values for each sample based on the definition of tumor mutation load (TMB). Copy number variation (CNV) data for the TCGA-LIHC cohort were downloaded from UCSC Xena (https://xenabrowser.net/datapages/), and we downloaded gene set data “c2.cp.kegg.v7.5.1.symbols.gmt”, “c2.cp.reactome.v7.5.1.symbols.gmt”, and “h.all.v7.5.1.symbols.gmt” from MsigDB database. Pathologic sections from 18 patients diagnosed with HCC between January 2016 and August 2019 were obtained from the Third Xiangya Hospital of Central South University. Among these patients, 9 had concurrent HBV infection, while the other 9 were non-HBV-infected individuals. Detailed clinical information of the patients can be found in Supplementary Table 1.

### Genetics and prognostic landscape construction for 31 disulfrgs

We used the “limma” R package to perform differential analysis between HCC and paraneoplastic samples in the “TCGA-LIHC” cohort, comparing mRNA expression differences between 31 disulfrgs in HCC and normal tissue. We used the “maftools” R package to analyze maf files and construct a mutational landscape of 31 disulfrgs in the TCGA-LIHC cohort in the form of waterfall plots. We analyzed the gain or loss of each gene based on the CNV data of the 31 disulfrgs. We divided the TCGA-LIHC cohort into high and low expression groups using the optimal cutoff value of the gene expression profile, and compared OS between the two groups using the “log-rank” method and the “Univariate Cox regression” method.

### The NMF clustering based on 31 disulfrgs

We clustered the TCGA-LIHC cohort by NMF based on the expression profiles of 31 disulfrgs [20], using the “brunet” method with 10 iterations. We chose the top point with the fastest cophenetic decline as the best classifier and classified all samples into different molecular subtypes. We used t-Distributed Stochastic Neighbor Embedding (t-SNE) to downscale and visualize the distribution of the 31 disulfrgs’ expressions among different disulfidptosis subtypes. We used Kaplan–Meier (KM) survival analysis to compare differences in OS and PFS among patients of different disulfidptosis subtypes, and used box line plots and feature heat maps to visualize the distribution of differential expression of 31 disulfrgs and clinical features among different disulfidptosis subtypes.

### Gene set variation analysis

We used the “GSVA” R package to perform gene set variation analysis (GSVA) between subtypes, using the gene set “c2.cp.kegg.v7.5.1.symbols.gmt”. The GSVA analysis was used to compare the variation of potential biological processes among different disulfidptosis subtypes.

### Differential analysis of the tumor microenvironment among different disulfidptosis subtypes

To assess the relative infiltration level of each immune cell, we employed the ssGSEA algorithm from the “GSVA” R package, which calculated the infiltration scores of 23 immune cells in the TME [21]. This analysis allowed us to evaluate the varying levels of immune cell infiltration among different disulfidptosis subtypes. Furthermore, we utilized the "ESTIMATE" R package to calculate the StromalScore, ImmuneScore, and ESTIMATEScore for each liver cancer sample. The StromalScore represents the stromal infiltration, the ImmuneScore represents the immune infiltration, and the ESTIMATEScore is the sum of the two, reflecting the overall infiltration abundance of stromal and immune components in the TME. To compare the infiltration levels of the 23 immune cell types between subtypes, as well as the StromalScore, ImmuneScore, and ESTIMATEScore, we conducted a differential analysis.

### Differential analysis and enrichment analysis between disulfidptosis subtypes

Using the “limma” R package, we performed differential analysis between different disulfidptosis subtypes, resulting in the identification of 1006 differential genes (DEGs). The screening criteria for DEGs were an FDR value less than 0.05 and a logFoldChange (logFC) greater than 1. Subsequently, we utilized the “clusterProfiler” R package to perform Gene Ontology (GO) and Kyoto Encyclopedia of Genes and Genomes (KEGG) functional enrichment analysis for the DEGs. This analysis helped identify significantly enriched biological processes (BP), cellular components (CC), molecular functions (MF), and signaling pathways [22].

### Random forest-based screening of subtype-specific genes

To identify subtype-specific genes, we employed the random forest (RF) algorithm using the “randomForest” R package. The default number of iterations is 100, considering the model robust enough when the RF algorithm built 500 decision trees. The Gini coefficient method was used to score the importance of the characteristic genes, and genes with a score greater than 4 were selected as the subtype-specific genes for further analysis and the construction of an artificial neural network (ANN) model.

### Construction of subtype differentiation ANN model

The subtype-specific genes were utilized to train an ANN model. These genes served as the input layer of the model. We employed the “0/1” assignment method to assign corresponding weight information and scores to the subtype genes. The “neuralnet” R package and “NeuralNetTools” R package were used to build the artificial neural network model. The model outputted subtype predictions, and the classification performance was assessed by plotting receiver operating characteristic (ROC) curves and calculating the area under the curve (AUC) using the "pROC" R package.

### Construction of weighted gene co-expression network analysis (WGCNA) and identification of key modules

To construct a co-expression network, we first selected DEGs among disulfidptosis subtypes and removed free individuals to obtain the input gene expression matrix. This matrix was then combined with the results of immune infiltration levels obtained using CIBERSORT to construct the WGCNA. Pearson correlation analysis was utilized to construct a weighted matrix, and power scatter plots were drawn to determine the best soft power (β) value. The weighted adjacency matrix was then constructed using the selected β value. The dynamic tree cutting method divided genes with similar expression levels into different modules, and gene trees were generated by hierarchical clustering based on the topological overlap matrix (TOM). Genes with similar expression profiles were grouped into modules, with each module containing at least 60 genes. By setting the threshold for module similarity at 0.25, we merged similar modules and identified the blue module as the key module related to immunosuppression.

### Construction and validation of disulfidptosis scoring system

By intersecting the 744 genes in the blue module with the 588 protein-coding genes among the 699 DEPGs, we obtained 503 significant genes (Siggs). These Siggs were further subjected to Least Absolute Shrinkage and Selection Operator (LASSO) regression using the “glmnet” R package for feature selection. LASSO regression compressed the regression coefficients and selected genes with non-zero coefficients for the next step of analysis. We performed multifactorial Cox regression to screen modeled genes and construct the disulfS. The modeled genes were named disulfidptosis potentially related genes (DPRGs). The disulfS was calculated using the formula: disulfS = h0(t) * exp (β1X1 + β2X2 + … + βnXn), where β represents the regression coefficient and h0(t) is the baseline risk function. Patients in the TCGA-LIHC cohort were divided into high and low disulfS groups based on the median. The correspondence between disulfrg clusters, gene clusters, disulfS grouping, and the survival of liver cancer patients in the TCGA cohort was visualized using sankey plots generated with the “ggalluvial” R package. We compared the differences in disulfS across disulfrg clusters and gene clusters using differential analysis. Additionally, KM survival analysis was conducted to compare the differences in OS between patients in the high and low disulfS groups in the TCGA training set, internal validation set, and external validation set. The predictive effect on PFS was also explored. ROC curves and calibration curves were used to assess the predictive accuracy of disulfS for 1-year, 3-year, and 5-year OS.

### Clinical subgroup analysis

Considering the importance of “Stage” and “Grade” as clinical subgroup characteristics in patients with HCC, we counted and compared the proportional distribution of these characteristics between the high and low disulfS groups. The results were visualized using stacked barplots. Furthermore, differential analysis was conducted to compare the differences in disulfS between subgroups of patients based on stage and grade. The impact of disulfS grouping on OS in these subgroups of patients was explored using KM survival analysis.

### DisulfS-based analysis of mutation, tumor stemness, and MSI

We employed the "Maftools" package to construct separate mutation landscapes for the high and low disulfS groups. Corresponding tumor mutation burden (TMB) values were calculated based on each TCGA liver cancer sample cohort [23]. Differential analysis was performed to compare the TMB differences between patients in the high and low disulfS groups, and correlation analysis was used to explore the relationship between disulfS and TMB. The tumor stemness index, mRNAsi, which represents the stemness level of tumor cells, was obtained from a previous study [24] based on mRNA expression profiles. Correlation analysis was used to investigate the association between disulfS and mRNAsi. Additionally, MSI analysis was conducted based on a previous study [23] to represent the level of microsatellite length change caused by mismatch repair mechanism (MMR) failure during DNA replication. Correlation analysis was employed to explore the association between disulfS and MSI.

### Disulf-based analysis of tumor immune microenvironment and immunotherapy efficacy

To evaluate the immune infiltration level in the tumor immune microenvironment (TIME) of LIHC, seven algorithms including “CIBERSORT,” “CIBERSORT-ABS,” “EPIC” [25], “MCPCOUNTER,” “QUANTISEQ” [26], “TIMER” [27], and “XCELL” [28] were used. Spearman correlation analysis was performed to explore the correlation between immune cell infiltration levels and disulfS obtained using the different algorithms. We compared the predicted immunotherapy responsiveness between patients in the high and low disulfS groups using differential analysis based on the IPS of two ICIs obtained from the TCIA database [29]. The IPS included ctla4_pos_pd1_pos, ctla4_neg_pd1_pos, ctla4_pos_pd1_neg, and ctla4_neg_pd1_neg. Higher IPS indicated greater responsiveness to the respective ICIs. Additionally, immune rejection (exclusion) scoring was obtained from the TIDE database (http://tide.dfci.harvard.edu/), and differential analysis was used to compare the difference in exclusion between patients in the high and low disulfS groups. We employed the GSE15654 immunotherapy cohort to investigate the predictive significance of disulfS for the PD-1 monoclonal antibody treatment population. Differential OS was compared across disulfS subgroups using KM survival analysis.

### Sorafenib sensitivity analysis

To predict the sensitivity of patients to sorafenib, the “pRRophetic” R package [30] was used to calculate the predicted half-inhibitory concentration (IC50) based on the Genomics of Drug Sensitivity in Cancer (GDSC) drug data source and gene expression profile data. Lower IC50 values indicated higher sensitivity to sorafenib. Differential analysis was performed to compare the predicted IC50 values for sorafenib between patients in the high and low disulfS groups, and correlation analysis was conducted to demonstrate the relationship between disulfS and the predicted IC50 values for sorafenib.

### Cell culture and real-time quantitative PCR (RT-qPCR)

Human normal hepatocytes (LX2) and hepatoma cells (MHCC97H, LM3) were obtained from Nanke Biotechnology Co. The cells were cultured in DMEM medium supplemented with penicillin G (100 mg/mL), streptomycin (100 mg/mL), and 10% fetal bovine serum (FBS; Gibco; USA) at 37 °C with 5% CO2. Logarithmically grown cells were used for subsequent experiments.

For RNA extraction, total RNA was isolated from the cells using the Fastern reagent (Invitrogen) according to the manufacturer's instructions. The purity of the extracted RNA was assessed spectrophotometrically (A260/A280 > 1.8). Subsequently, reverse transcription followed RT-qPCR was performed using an SYBR Green PCR Master Mix. The PrimeScript RT Reagent Kit (TaKaRa, Shiga, Japan) was used to reverse transcribe 1 μg of total RNA into cDNA. The relative RNA expression was determined using the 2-△△Ct method, with GAPDH serving as the internal loading control for normalization. The following primer sequences were used in this study: SLC7A11 (f: GCGTGGGCATGTCTCTGAC, r: GCTGGTAATGGACCAAAGACTTC) and SLC2A1 (f: ATTGGCTCCGGTATCGTCAAC, r: GCTCAGATAGGACATCCAGGGT).

### Cell viability assay and reagents

A cell viability assay was performed using the following reagents: 5 μM Necrostatin-1 (Nec-1), 5 μM Necrostatin-2 (Nec-2), 25 μM chloroquine (CQ), 10 μM Z-VAD-FMK, and 100 μM Deferoxamine mesylate (DFOM) [15]. The control group was treated with an equal volume of DMSO. After 8 h of treatment, the old culture medium was discarded and replaced with complete medium DMEM containing 10% CCK8 reagent (Biosharp, BS350B). The absorbance at 450 nm was measured using a BIOTEK ELX800 plate reader. The following reagents were obtained from MedChemExpress: Ferr-1(HY-100579), Nec-1(HY-15760), Nec-2(HY-14622), CQ(HY-17589A), Z-VAD-FMK(HY-16658B), DFOM(HY-B0988).

### Fluorescence staining of actin filaments

2 × 10^5^ MHCC97H and LM3 cells in logarithmic growth phase were inoculated into 6-well plates with cell crawlers (Biosharp, BS-24-RC) and allowed to crawl for 24 h. After cell crawling, the cells were treated with either DMSO or BAY-876 in different wells for 8 h. The old medium was discarded, and the cells were washed with PBS and fixed with 4% paraformaldehyde at room temperature. The fixed cells were permeabilized with osmotic buffer (PBS containing 0.5% Triton X-100), washed with PBS, and incubated with 100 μM actin staining [Phalloidin-iFluor 555 (ab176756)] in the dark at room temperature for 30 min. After washing with PBS, the cells were incubated with DAPI working solution in the dark at room temperature for 10 min. The cells were then washed with PBS and imaged using a Zeiss inverted fluorescence microscope (Vert A1).

### Immunohistochemical staining and integrated optical density (IOD) analysis

Paraffin sections were incubated at 60 ℃ for a minimum of 60 min. Subsequently, the sections were deparaffinized using xylene and hydrated with varying concentrations of ethanol (10 min in 100% ethanol, followed by another 10 min in 100% ethanol, 5 min each in 95%, 90%, and 85% ethanol, and finally, 5 min in 70% ethanol). The sections were thoroughly rinsed with phosphate-buffered saline (PBS). After washing with PBS, endogenous catalase was used to block non-specific staining. Following blocking, the sections were washed with PBS three times before overnight incubation for four days with the respective primary antibodies. Afterward, the sections were washed with PBS three times and subsequently incubated at room temperature for 60 min with HRP-labeled secondary antibodies specific to the corresponding genus. Next, an appropriate amount of biotin substrate was added, and the reaction was carried out at room temperature for 30 min, followed by three additional washes with PBS.Upon completion of the reaction, 1 × DAB chromogenic solution was added to the sections. Hematoxylin re-staining was performed after the chromogenic development was completed. Finally, the tissues were dehydrated and sealed using a transparent agent. Photomicrographs were captured at a magnification of 200 × using a Zeiss inverted fluorescence microscope (Vert A1). IOD analysis was conducted using Image J software. The immunohistochemistry score was calculated as IOD divided by the collection area.The following reagents were obtained from Abiowell Biotechnology: ADH4 (AWA48164), NEIL3 (AWA52502), and STC2 (AWA48163). Additionally, CFHR3 (16583-1-AP) and MMP1 (10371-2-AP) were sourced from Proteintech.

### PBMC were obtained and co-cultured with HCC cell lines

PBMC cells (CP-H182) supplied by Prosperity Life Sciences Co. were co-cultured with HCC cell lines. The co-culture conditions included RPMI-1640 culture medium containing 10% FBS and 1% penicillin/streptomycin (100 U/mL and 100 μg/mL, respectively). 2 × 10^5^ LM3 or MHCC97H cells (effector cells, E) in logarithmic growth phase were inoculated into the lower chamber of a 12-well Transwell system with a pore size of 0.4 μm. After 24 h of culture, PBMC cells (target cells, T) at a concentration of 2 × 10^6^ cells/mL were inoculated into the corresponding upper chamber. The co-culture system was divided into three groups: a blank group with only E cells in the lower chamber and RPMI-1640 complete medium in the upper chamber; a control group with E cells in the lower chamber and T cells in the upper chamber; and an experimental group with E cells in the lower chamber treated with 5 μM BAY-876 and T cells in the upper chamber. After 48 h of co-culture, the culture medium from the upper chamber of each group was collected, centrifuged, and stored at – 80 ℃ for subsequent cytokine detection.

### Enzyme-linked immunosorbent assay (ELISA)

Concentrations of TNF-α, IL-6, and IL-1β were measured in the culture supernatant as indicators of PBMC cell activation. Cytokine concentrations were measured using commercially available ELISA kits targeting human cytokines. The concentrations of cytokines in the supernatants were assayed according to the guidelines provided by the manufacturers: IL-1β and IL-6 (Servicebio, Wuhan, CHINA), and TNF-α (Elabscience, Wuhan, CHINA). Each kit targeted an individual cytokine, and the detection level for all cytokines was 30 pg/mL. The coefficients of variation for the ELISA assays for cytokines were 2% or less of the median.

### Statistical analysis

All statistical and bioinformatics analyses were performed using the R language (version 4.2.1) and the perl language. The perl language was primarily used for data batch cleaning. Differential analysis was conducted using the "limma" R package, unless otherwise specified. In the bioinformatics section, comparisons between two groups were assessed using the Wilcoxon test, while the Kruskal–Wallis test was employed for comparisons involving more than two groups. Kaplan–Meier survival analysis and log-rank test were used to compare the prognosis of patients in different groups. Statistical significance was defined as a two-tailed p < 0.05 for all analyses.

## Results

### Differential expression of disulfrgs, genetic alterations, and prognostic significance

The study's flow chart is depicted in Graphical abstract. Initially, we identified 31 disulfrgs (*SLC7A11, SLC3A2, RPN1, NCKAP1, RAC1, WASF-2, CYFIP1, ABI2, BRK1, SLC2A1, GYS1, OXSM, NDUFS1, NDUFA11, NUBPL, LRPPRC, PRDX1, FLNA, FLNB, MYH9, MYH10, TLN1, ACTB, CD2AP, INF2, ACTN4, PDLIM1, IQGAP1, DSTN, CAPZB,* and *MYL6*) for further analysis. Subsequently, 371 samples from the TCGA database were included in the analysis, and their basic information is provided in Supplementary Table 2. Differential expression analysis of disulfrg in cancer and paraneoplastic tissues was conducted using the TCGA-LIHC database. The results demonstrated that all 27 disulfrgs, except for *NDUFS1, NUBPL, MYH10,* and *IQGAP1* genes, exhibited significantly higher expression in tumor tissues (Supplementary Fig. 1A). Mutation status and copy number variation frequencies of disulfrgs were also examined. We observed that 54 samples (14.56%) out of the 371 samples had disulfrg mutations. Notably, *TLN1, FLNB,* and *IQGAP1* showed a high mutation frequency of 2% (Supplementary Fig. 1B). Furthermore, we found that the copy number deletion frequencies of *SLC2A1, CAPZB, PRDX1, NDUFA11, PDLIM1, WASF2, INF2,* and *MYH10* were significantly higher compared to the frequencies of copy number increase. Interestingly, GYS1, RPN1, and DSTN exclusively exhibited copy number deletion variants (Supplementary Fig. 1C). Moreover, we investigated the chromosomal localization of copy number variants in HCC patients. The results revealed that copy number variants of *CAPZB, WASF2,* and *SLC2A1* were localized on chromosome 1, *LRPPRC, NCKAP1, ABI2,* and *BDUFS1* on chromosome 2, and *BRK1, OXSM, FLNB,* and *RPN1* on chromosome 3. The copy number variants of other disulfrgs were scattered (Supplementary Fig. 1D).

Subsequently, we assessed the prognostic significance of disulfrgs in HCC patients using univariate Cox regression. The results indicated that all remaining 19 genes in the disulfrgs set were significantly associated with OS, except for *CYFIP1, NDUFS1, NDUFA11, NUBPL, FLNA, FLNB, MYH9, MYH10, TLN1, PDLIM1, IQGAP,* and *MYL6* (Supplementary Fig. 2A). We selected the 9 disulfrgs most associated with disulfidptosis, as described in the article [16], for Kaplan–Meier curve plotting and presentation (Supplementary Fig. 2B). The outcomes revealed that patients with high expression levels of these nine genes exhibited worse prognosis. Thus, all of them were considered prognostic risk factors, which coincided with the differential expression pattern of genes in cancer versus paracancer. In other words, genes highly expressed in tumor tissues relative to normal tissues were associated with a worse prognosis when analyzing survival in tumor tissues.

### Differentiation of molecular typing based on NMF and comparison between different subtypes

**Fig.1:**
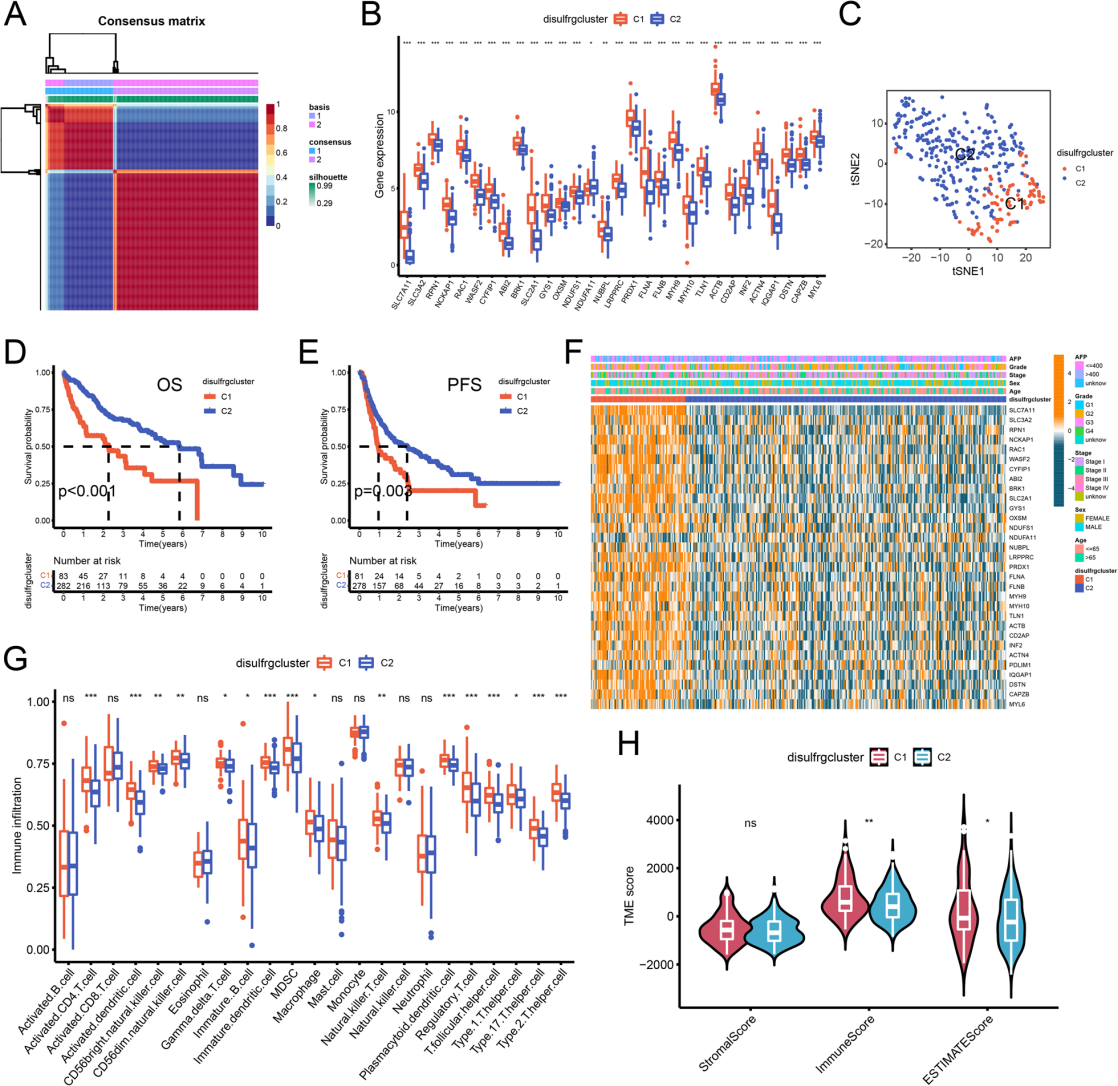
Identification and exploration in survival, clinical features, immune cell infiltration and TME scores of two disulfidptosis subtypes. **A** NMF clustering divides HCC samples into two clusters (k = 2) based on 31 disulfrgs. **B** Differential expression of disulfrgs between the two subtypes. **C** tSNE descending dimension analysis of the two subtypes. **D**, **E** OS and PFS curves for the two subtypes of patients with HCC. **F** Difference distribution of clinicopathological features and disulfrgs expression among the two subtypes. **G** Difference analysis of 23 immune infiltrating cells levels between the two subtypes. **H** Analysis of differences in TME scores between the two subtypes. *p < 0.05, **p < 0.01, ***p < 0.001; ns: not statistically different

NMF typing of HCC patients was performed based on the expression levels of 31 disulfrgs. The optimal rank value was determined based on the fastest decrease in cophenetic points, and for this study, a rank = 2 was chosen to classify HCC patients into two disulfrg clusters, C1 and C2 (Fig. 1A). The analysis of disulfrgs expression levels in the two disulfidptosis subtypes revealed that all 30 disulfrgs exhibited significant differential expression, except for *PDLIM1*. Moreover, except for *NDUFA11*, all 29 disulfrgs were found to be lowly expressed in subpopulation C2 (Fig. 1B). Subsequently, a t-distributed Stochastic Neighbor Embedding (tSNE) analysis was conducted between C1 and C2 subgroups, followed by a comparison of the differences in disulfrgs expression levels, survival, clinical characteristics, immune cell infiltration levels, and TME scores between the two clusters. The tSNE plot demonstrated clear discrimination between the two subgroups (Fig. 1C). Kaplan–Meier survival analysis demonstrated significant differences in OS and PFS between the different disulfidptosis subtypes. Patients with HCC in the C2 subpopulation exhibited a better prognosis (Fig. 1D, E). Additionally, a heat map illustrating the differential expression of clinical characteristics among different subgroups after NMF clustering was constructed based on the age, gender, clinical stage, histological grade, and alpha-fetoprotein (AFP) level of HCC patients in TCGA (Fig. 1F).

To gain further insights into the reasons for the prognostic differences between different disulfidptosis subtypes, we compared the infiltration levels of 23 immune cells between the clusters using the single-sample Gene Set Enrichment Analysis (ssGSEA) algorithm. The analysis revealed that the infiltration levels of immunosuppressive cells such as regulatory T cells (Tregs), T follicular helper (Tfh) cells, and myeloid-derived suppressor cells (MDSC) were lower in the C2 subpopulation (Fig. 1G). Moreover, we compared the TME scores between different subpopulations and estimated the ratio of immune stromal components in the TME for each sample using the ESTIMATE algorithm. The results indicated that the ESTIMATE score was significantly lower in the C2 subpopulation compared to the C1 subpopulation (Fig. 1H). Additionally, GSVA was performed to compare between disulfidptosis subtypes. The results revealed upregulation of linoleic acid metabolism and glycine/serine/threonine metabolic pathways in the C2 subpopulation, as determined by the KEGG reference gene set. In the Reactome reference gene set, pathways related to plasma lipoprotein and cholinesterase remodeling, liposome assembly, and protein amine terminal clearance and transport were upregulated in the C2 subpopulation. Furthermore, the hallmark reference gene set indicated upregulation of xenobiotic metabolism and bile acid metabolism pathways in the C2 subgroup (Supplementary Fig. 3). These findings suggest that patients with HCC in the C2 subgroup exhibit a better clinical prognosis, which may be closely associated with the lower levels of immunosuppressive cell infiltration, lower ESTIMATE score, and upregulation of metabolism-related pathways. Moreover, patients in the C2 subgroup may be more responsive to immunotherapy.

### Enrichment analysis of DEGs and construction of RF and ANN model

To further explore the underlying biological behavior associated with different prognoses between C1 and C2 subgroups, differential analysis of gene expression profiles was performed on patients from the two disulfidptosis subtypes. This analysis identified 1006 differentially expressed genes (DEGs) between the subgroups. A volcano plot was constructed to visualize the DEGs (Supplementary Fig. 4A). Subsequently, GO and KEGG enrichment analyses were conducted to determine the potential functions and pathways associated with the DEGs. The GO analysis revealed that the DEGs were mainly involved in the regulation of intracellular immune effector processes in terms of biological processes (BP). In terms of cellular components (CC), the DEGs were mainly associated with collagen-containing extracellular matrix and cytoplasmic vesicle components. The molecular function (MF) analysis suggested that the DEGs were involved in protein binding and catalytic activity. The KEGG enrichment analysis indicated upregulation of complement and coagulation cascades, drug metabolism-cytochrome P450, ECM-receptor interactions, and glycolysis/gluconeogenesis pathways in the C2 subpopulation (Supplementary Fig. 4B–D).

Next, the 1006 DEGs were used in a RF classification to identify key genes that distinguish the two disulfidptosis subtypes. The RF model employed 500 decision trees as parameters based on the correlation plot between the number of RF branches and the model error. The analysis identified *SLC7A11* as the most significant gene, followed by *SLC2A1, ADAM9, ITGAV,* and *PFKP* (Supplementary Fig. 5A, B). Subsequently, an artificial neural network model was constructed based on the expression matrix of these five genes and the two subgroups. The ANN model consisted of five input layers, four hidden layers, and two output layers (Supplementary Fig. 5C). Cross-validation results were represented by ROC curves, which demonstrated that *SLC7A11*, *SLC2A1, ADAM9, ITGAV,* and *PFKP* were the most characteristic genes for distinguishing between C1 and C2 subgroups. The model constructed based on these genes exhibited reliability, with an AUC value of 0.951 (95%CI 0.923–0.972) (Supplementary Fig. 5D).

Taken together, the clustering and typing of HCC patients into two disulfrg clusters using NMF based on the expression levels of 31 disulfrgs is highly reliable and distinguishable. The phenotypic differences between the two disulfrg clusters are primarily attributed to the differential enrichment of immune and metabolic pathways.

### WGCNA analysis and prediction scoring system construction

**Fig. 2:**
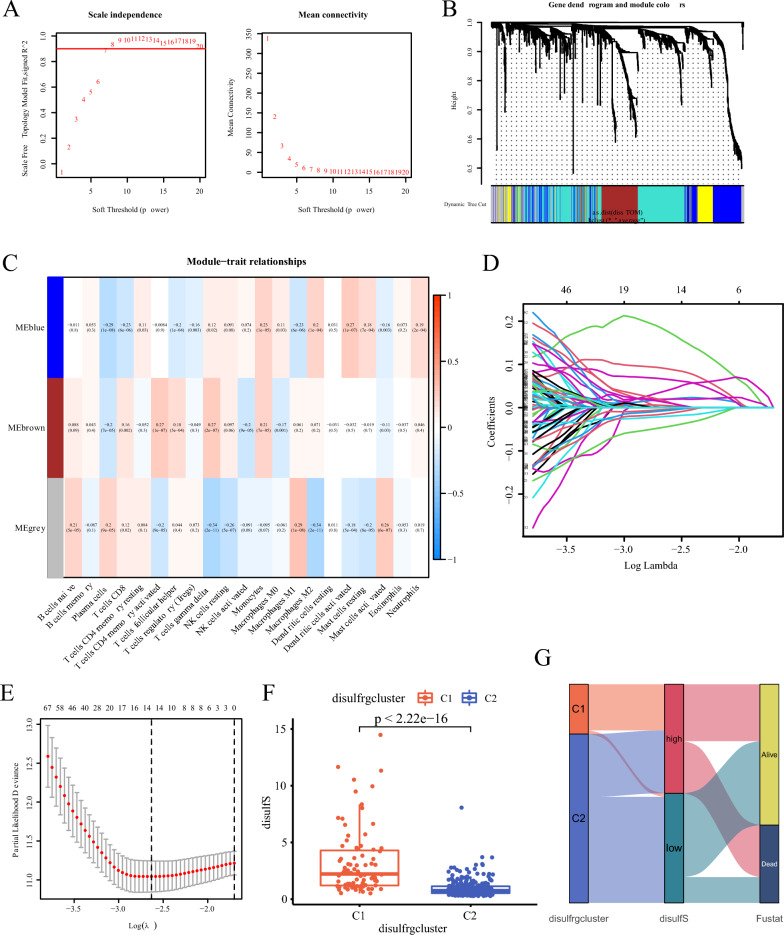
Construction of disulfS prediction scoring system. **A** The selection of the best-fit power value, softpower (β) = 7, was based on fit index and average connectivity. **B** A hierarchical clustering tree using the correlation TOM matrix between DEGs. **C** A module-immune cell correlation heat map. **D** 10 × cross-validation of lasso regression model coefficients for DEPGs. **E** Stability analysis of lasso regression model. The stability of the model was the highest when the number of genes was 14. **F** Distribution of disulfS among different disulfidptosis subtypes. **G** The Sankey diagram provided a visual representation of the correspondence between different groups of patients

To gain deeper insights into the potential association between different disulfidptosis statuses and immune effects in HCC patients, we constructed a WGCNA network. This network integrated the gene expression matrix of the 1006 DEGs with the results of immune infiltration levels obtained from CIBERSORT. The selection of the best-fit power value, softpower (β) = 7, was based on fit index and average connectivity (Fig. 2A). We subsequently constructed a hierarchical clustering tree using the correlation TOM matrix between genes, where different branches and colors represented distinct gene modules (Fig. 2B). After merging similar modules, we plotted a module-immune cell correlation heat map, focusing on the blue module (Fig. 2C). We conducted an univariate Cox analysis on the 1006 differentially expressed genes (DEGs) obtained earlier, identifying 619 differential prognostic genes (DEPGs) at a significance level of p < 0.05. Intersection analysis between this module and the 588 protein-coding genes among the 619 DEPGs yielded 503 Siggs. Further, we divided the TCGA liver cancer patients into a training group (n = 221) and an internal validation group (n = 143) at a ratio of 6:4. The training group included the 503 Siggs for lasso regression and multifactorial Cox regression analysis, leading to the identification of five DPRGs: *NEIL3, MMP1, STC2, ADH4,* and *CFHR3*. Based on these five genes, we constructed the disulfS to evaluate the disulfidptosis status of each patient (Supplementary Table 3 and Fig. 2D, E). The disulfS was calculated as follows: disulfS = 1.006 * exp [*NEIL3* × (0.346) + *MMP1* × (0.147) + *STC2* × (0.189) + *ADH4* × (− 0.056) + *CFHR3* × (− 0.112)], gene symbol represents the expression level of the gene. By using disulfS, we calculated the risk score for patients with HCC and divided them into low- and high-risk groups based on the median score. Comparison of disulfS revealed significantly lower scores in groups C2 compared to groups C1 in terms of disulfrgcluster (Fig. 2F). The Sankey diagram provided a visual representation of the prediction model construction process, reflecting the correspondence between disulfrgcluster, disulfS grouping, and survival in patients with HCC (Fig. 2G).

### Predictive value validation of the disulfidptosis scoring system

To assess the predictive power of the disulfS model, we generated differential expression heatmaps and disulfS risk curves for the five DPRGs in the TCGA-train, TCGA-test, and TCGA-all cohorts (Supplementary Fig. 6A–C). These results revealed that *NEIL3, MMP1,* and *STC2* were highly expressed in the high-risk group, while *ADH4* and *CFHR3* showed low expression. Moreover, as the disulfS increased, the number of patient deaths also increased. These findings indicate that the constructed disulfS model effectively stratifies HCC patients into high- and low-risk groups, with *NEIL3, MMP1,* and *STC2* serving as prognostic risk factors. Univariate analysis demonstrated a significant association between OS in HCC patients and both stage and the disulfS model (Supplementary Fig. 6D, p < 0.001). Multivariate analysis further confirmed that both stage (HR = 1.559, p < 0.001) and the disulfS model (HR = 1.223, p < 0.001) independently predicted OS in HCC patients (Supplementary Fig. 6E).

**Fig. 3:**
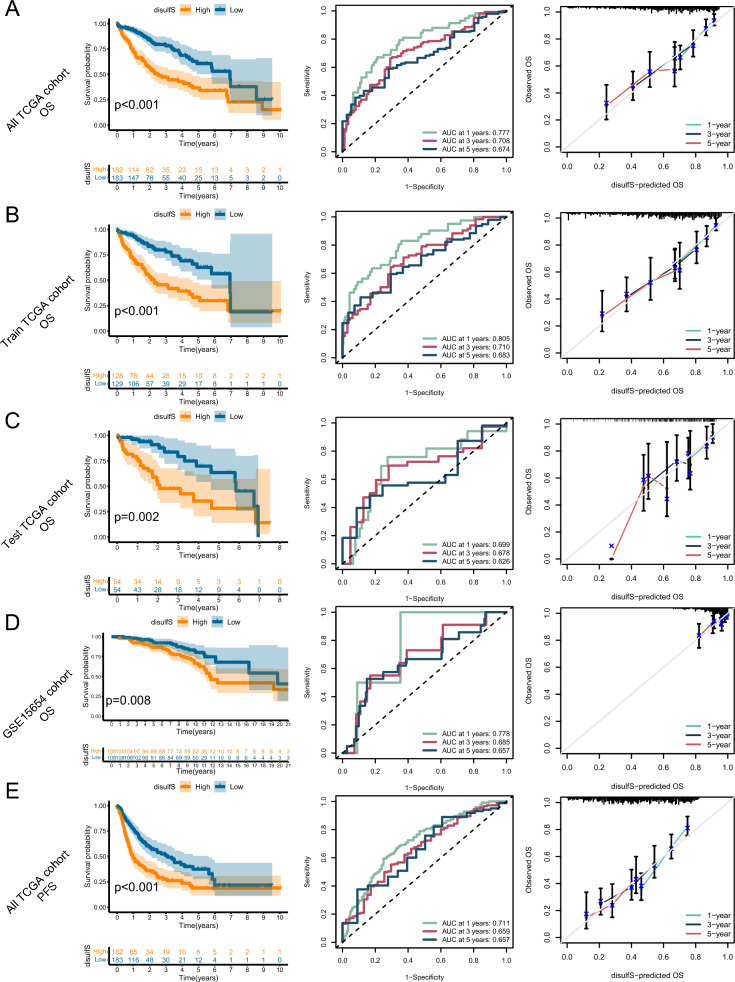
Prediction effect validation of disulfS in different cohorts. **A** KM plots of OS differences between high and low disulfS groups in the TCGA-all cohort, ROC curves, and calibration curves. **B** KM plots of OS differences between high and low disulfS groups in the TCGA-test cohort, ROC curves, and calibration curves. **C** KM plots of OS differences between high and low disulfS groups in the TCGA-train cohort, ROC curves, and calibration curves.** D **KM plots of OS differences between high and low disulfS groups in the GSE15654 cohort, ROC curves, and calibration curves. **E** KM plots of PFS differences between high and low disulfS groups in the TCGA-all cohort, ROC curves, and calibration curves

To evaluate the ability of the disulfS model to guide prognosis in HCC patients, we performed KM curve analysis for OS in the high- and low-risk disulfS groups in the TCGA-all, TCGA-train, TCGA-test, and GSE15654 cohorts. Additionally, we validated the accuracy of the disulfS model using ROC curves, calibration curves, and accuracy assessments (Fig. 3A–D). The results demonstrated that the disulfS model effectively differentiated the OS prognosis of HCC patients, with worse outcomes observed in the high-risk disulfS group. Encouragingly, the ROC curves, calibration curves, and accuracy assessments indicated excellent performance and consistency of the disulfS model in predicting 1-, 3-, and 5-year OS in HCC patients across internal and external validation cohorts. Furthermore, we assessed the predictive ability of the disulfS model for PFS in HCC patients, revealing similarly efficient results as observed for OS prediction (Fig. 3E).

Given the importance of stage and grade as clinical subgroup characteristics in HCC patients, we investigated the ability of the disulfS model to guide the prognosis in different clinical subgroups. We compared the proportional distribution of disulfS among different grade and stage subgroups and analyzed the predictive capacity of high and low disulfS for OS in these subgroups. The results showed that the differences in disulfS were also significant between grade G1/G2 and grade G3/G4 patients (Supplementary Fig. 7A, B) and between stage I/II and stage III/IV patients (Supplementary Fig. 7C, D). Importantly, the disulfS exhibited excellent predictive ability for OS in various clinical subgroups (Supplementary Fig. 7E–H).

In summary, our construction of the disulfS model serves as an independent prognostic factor for OS in HCC patients. It effectively stratifies patients into high- and low-risk groups, with higher-risk patients exhibiting higher grade, later stage, and worse prognosis.

### Correlation of disulfS with MSI, tumor stemness index (mRNAsi), TIME, and gene mutation frequency (GMF)

**Fig. 4:**
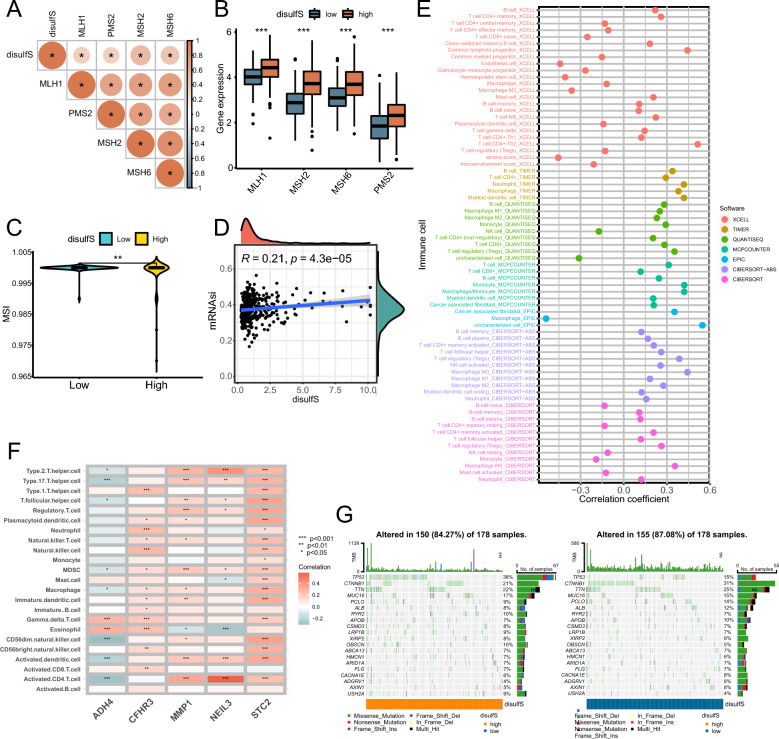
Correlation of disulfS with MSI, mRNAsi, TIME, and GMF. **A** Correlation distribution of disulfS with mismatch repair genes. **B** Boxplot to show differences of mismatch repair genes between high and low disulfS groups. **C** Violin plots to show differences of MSI scores from the TIDE database between high and low disulfS groups. **D** Correlation scatter plot of disulfS and mRNAsi. **E** Correlation of disulfS with immune cell infiltration level calculated by 7 immune infiltration algorithms. **F** Correlation heatmap of 5 DPRGs and immune cell infiltration level calculated by ssGSEA. **G** Waterfall plot of GMF for high and low disulfS groups. *p < 0.05, **p < 0.01, ***p < 0.001

To investigate the factors underlying the differentiation of HCC patients based on disulfS, we analyzed its relationship with MSI, mRNAsi, TIME, and GMF. Our findings revealed significant positive correlations between disulfS and four mismatch repair genes (Fig. 4A), patients with high disulfS had higher expression of mismatch repair genes and lower MSI (Fig. 4B, C). Furthermore, disulfS showed a significant positive correlation with mRNAsi (Fig. 4D). We also used seven common immune infiltration analysis algorithms (CIBERSORT, CIBERSORT-ABS, EPIC, MCPCOUNTER, QUANTISEQ, TIMER, and XCELL) to assess the association between disulfS and immune cells in TIME (Fig. 4E). Notably, the four DPRGs (*NEIL3, MMP1, STC2,* and *CFHR3*) in the disulfS model were positively correlated with the levels of most immune infiltrating cells, except for *ADH4* (Fig. 4F). Additionally, we compared the GMF between the high and low disulfS groups, revealing a higher frequency of mutations in the common driver gene *TP53* of HCC in the high disulfS group (Fig. 4G).

### Role of disulfS in predicting the efficacy of immunotherapy and Sorafenib

**Fig. 5:**
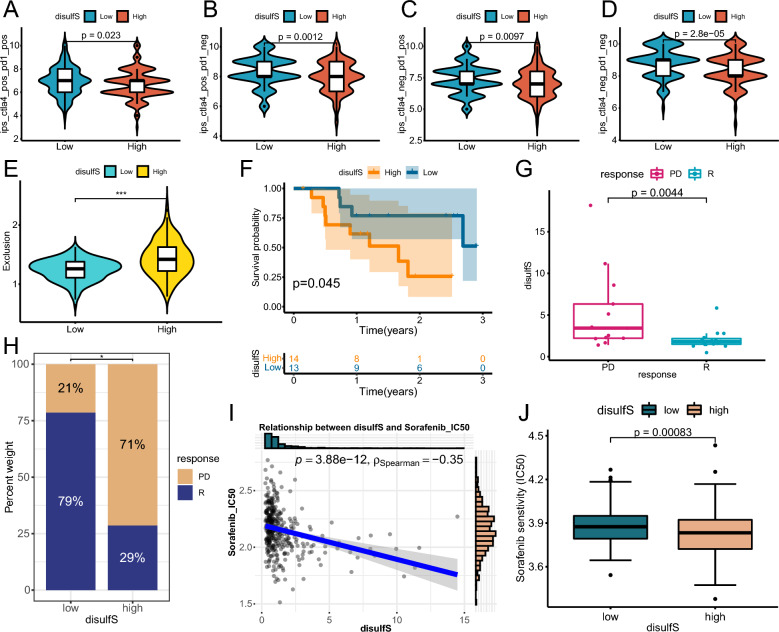
Role of disulfS in predicting the efficacy of immunotherapy and sorafenib. **A**–**D** Violin plots to show differences of the four IPS between high and low disulfS groups. **E** Violin plot to show differences of Exclusion scores between high and low disulfS groups. **F** KM curve based on disulfS predicting OS in the GSE78220 cohort. **G**, **H** Exploring the association between disulfS and different immunotherapy response states in the GSE78220 cohort. **I** Correlation scatter plot of disulfS and predicted sorafenib IC50 value. **J** Boxplot to show differences of predicted sorafenib IC50 value between high and low disulfS groups. *p < 0.05

To assess whether disulfS can guide immunotherapy and drug therapy in clinical HCC patients, we analyzed the IPS of two ICIs, anti PD-1 and anti CTLA-4, using TCIA data. We compared the differences in IPS between the high and low disulfS groups across immunotherapy groups. The results demonstrated significantly higher IPS in the low disulfS group compared to the high disulfS group (Fig. 5A–D). We further utilized the TIDE database to score patients in the high and low disulfS groups for "Exclusion" and found higher Exclusion scores in the high disulfS group (Fig. 5E). Moreover, we validated the efficacy of immunotherapy in the GSE15654 cohort, consisting of 27 samples treated with anti PD-1. The results indicated that the disulfS model served as a reliable predictor of patient OS, patients who responded to immunotherapy had lower disulfS (Fig. 5F–H). These findings suggest that the disulfS model holds potential for predicting the effectiveness of immunotherapy in patients. Lastly, we compared the drug sensitivity of the high and low disulfS groups to sorafenib, a first-line treatment for clinical HCC patients. The results revealed a significant negative correlation between the disulfS model and the IC50 value of sorafenib, indicating that the high disulfS group had a better treatment response to sorafenib. Therefore, in clinical settings, immunotherapy might be a preferable option for patients with a low disulfS rather than targeted therapy with sorafenib.

### Confirmation of disulfidptosis in HCC and its association with immune response

**Fig. 6:**
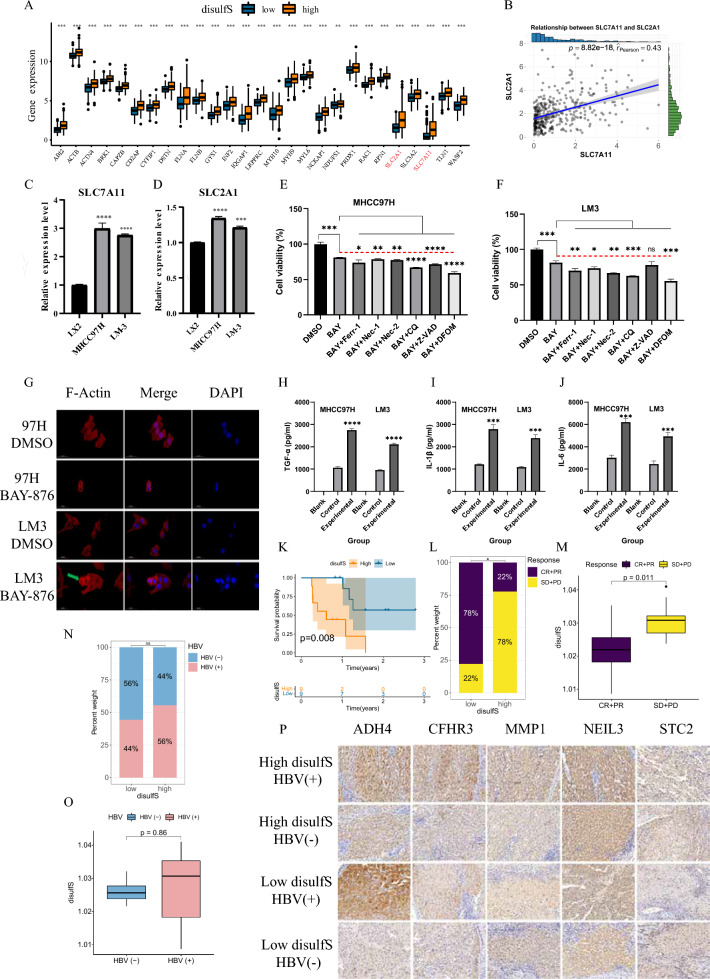
Confirmation of the presence of disulfidptosis in HCC and its association with immune response and the effect of disulfS in a clinical cohort. **A** Box line plot of differential expression of 31 disulfrgs between high and low disulfS groups. **B** Scatter plot of correlation between SLC7A11 and SLC2A1 gene expression. **C**, **D** RT-qPCR validation of mRNA expression of SLC7A11 and SLC2A1 in three cell lines, LX2, MHCC97H and LM-3. SLC7A11 highly expressed MHCC97H cells (**E**) and LM3 cells (**F**) were cultured in glucose-normal and glucose-suppressed medium with or without FERR-1, Z-VAD, NEC-1, NEC-2, CQ, and DFOM for 8 h. The dashed red line represents the proliferative viability of cells under glucose-normal conditions, serving as the reference line for other treatments. **G** Fluorescence staining of F-actin was performed using oncolytic ethidium in MHCC97H and LM3 cells cultured for 8 h under glucose-normal and glucose-suppressed conditions. Levels of cytokines TGF-α (**H**), IL-1β (**I**), and IL-6 (**J**) were assayed in the cell supernatants of the co-culture model involving HCC cells and PBMCs. **K** Kaplan–Meier (KM) curves were analyzed to assess progression-free survival (PFS) in 18 patients. The proportional distribution of disulfS among different treatment responses (**L**) and HBV expression (**N**). Boxplots depict the differences in treatment response (**M**) and HBV expression (**O**) between the high and low disulfS groups. **P** Immunohistochemical results from four patients are presented. *p < 0.05, **p < 0.01, ***p < 0.001, ****p < 0.001

Based on the results obtained from RF and ANN modeling, we identified five characterized genes (*SLC7A11, SLC2A1, ADAM9, ITGAV,* and *PFKP*) that distinguish HCC patients into different subgroups of disulfide clusters (Supplementary Fig. 5). Notably, *SLC7A11 and SLC2A1* were identified as key genes for disulfide metabolism disorders in tumor cells [16] and exhibited a high predictive value for OS (Supplementary Fig. 2B). Therefore, we focused our investigation on these two genes. Firstly, we compared the differential expression levels of 31 disulfides between the high and low disulfidptosis subtype, particularly emphasizing the significant upregulation of *SLC7A11* and *SLC2A1* in the high disulfidptosis subtype (Fig. 6A). Subsequently, correlation analysis revealed a significant positive correlation between the expression of *SLC7A11* and *SLC2A1* (Fig. 6B). To validate the expression levels of these two signature genes at the cellular level, we performed qPCR experiments. The results confirmed higher expression of *SLC7A11* and *SLC2A1* in HCC cells (MHCC97H, LM3) compared to human normal hepatocytes (LX2) (Fig. 6C), consistent with the findings in Fig. 2A.

Next, to corroborate the presence of disulfide death within HCC, we followed a previous study's methodology and employed various known cell death inhibitors in combination with the GULT1 inhibitor BAY-876 to treat the *SLC7A11* highly expressed MHCC97H and LM3 HCC cell lines under glucose starvation conditions. Interestingly, the known cell death inhibitors, including iron death inhibitors (Ferr-1 and DFOM), apoptosis inhibitor (Z-VAD-FMK), necrosis inhibitors (NEC-1 and -2), and autophagy inhibitor (CQ), were unable to reverse cell death compared to the DMSO group (Fig. 6E, F). Thus, we tentatively concluded that disulfide death may exist in HCC. Additionally, we stained actin filaments of MHCC97H and LM3 cells treated with BAY-876 for 8 h to determine whether this mode of cell death was associated with cytoskeletal dynamics. It was noteworthy that glucose starvation induced significant changes in cellular morphology, characterized by cytoconstriction and F-actin contraction (Fig. 6G). Our previous biosignature analysis suggested that disulfide death might be associated with the immunotherapeutic response of HCC patients. To demonstrate this, we stimulated PBMC cells with HCC cells undergoing disulfide death in a co-culture system, aiming to observe the secretion of immunomodulatory factors such as TNF-α, IL-1β, and IL-6. Excitingly, ELISA analysis revealed significantly higher levels of these cytokines in the experimental group’s supernatant serum compared to the control group (Fig. 6H, I). Thus, we determined that disulfide death can occur in HCC cells highly expressing *SLC7A11* under glucose starvation conditions and may play a potential role in immunosensitization.

### Confirmation of disulfS effect in clinical cohort

We were particularly interested in examining whether the pre-constructed disulfS signature could be applied in a clinical setting and whether it exhibited differential prognostic effects in HBV ( +) and HBV (−) patients. To investigate this, we collected 18 pathologic sections from patients diagnosed with HCC at Xiangya Third Hospital of Central South University. Among these samples, 9 were HBV ( +) and 9 were HBV (−). We performed immunohistochemical staining for the five key disulfS construct genes (NEIL3, MMP1, STC2, ADH4, CFHR3) in the 18 samples and conducted IOD analysis to relatively quantify the expression levels of these five genes among different patients.Subsequently, we categorized the 18 patients into high disulfS and low disulfS groups. Kaplan–Meier curve analysis revealed that patients with high disulfS had lower progression-free survival (PFS) and inferior treatment efficacy (Fig. 6K–M). Importantly, we observed no significant difference in the distribution of disulfS between HCC patients with HBV ( +) and HBV (−) (Fig. 6N, O). Finally, we selected the immunohistochemical results of four patients for presentation.

## Discussion

In recent years, the incidence of HCC has been increasing, and it is projected to exceed 1 million cases by 2025 [31]. While advancements in diagnostics and treatments have improved outcomes for early-stage HCC patients, the overall prognosis for HCC remains poor [32]. Conventional therapies such as surgical resection, radiofrequency ablation, and transarterial chemoembolization (TACE) are commonly used for early-stage HCC patients [33–35]. However, treatment options for patients with advanced stages have been limited to palliative care. The emergence of immunotherapy has significantly improved the prognosis of advanced HCC patients, with studies demonstrating the close relationship between immune cell composition and treatment response in HCC [36–38].

Disulfidptosis, a novel form of cell death, holds great potential in tumor development and immunotherapy. Previous studies have shown that disulfide bond polymerization in mitochondria can alter tumor progression [39]. The use of disulfide-bonded polymers as drug carriers has shown promise in enhancing the effectiveness of tumor chemotherapy by modulating redox levels [40]. Additionally, molybdenum disulfide (MoS2) combined with a metal–organic backbone has been explored for targeted cancer therapeutic diagnostics [41]. Liu et al. proposed that the accumulation of disulfide in tumor cells with high expression of *SLC7A11* could induce disulfide stress, leading to cell disintegration and death, opening up new avenues for tumor treatment [15].

Traditionally, HCC classification has been based on the pathological characteristics of cancer cells. However, several studies have suggested that HCC subtypes based on distinct characteristics can provide valuable clinical insights and prognostic information [42, 43]. In this study, we classified HCC patients into two disulfidptosis subtypes (C1 and C2) based on the expression profiles of 31 disulfrgs using the NMF algorithm. These subtypes exhibited different biological and clinical features. Notably, the C2 subtype, characterized by low expression of most disulfrgs, demonstrated better OS and PFS prognosis. The low disulfidptosis subtype was associated with improved outcomes. Further analysis revealed that the low disulfidptosis subtype had lower levels of immunosuppressive cell infiltration, including Treg, Tfh, MDSC, and ESTIMATE score. Treg and Tfh cells have been implicated in maintaining an immunosuppressive tumor microenvironment that inhibits the therapeutic effects of PD-1 [44]. MDSCs suppress T cell responses and possess immunosuppressive effects [45]. The ESTIMATE score represents tumor purity, and lower tumor purity generally correlates with better prognosis [46]. Moreover, the low disulfidptosis subtype showed activation of the glycine/serine/threonine metabolic pathway, which promotes glutathione synthesis and tumor cell killing [47]. In contrast, pathways related to intracellular immune effects and the PI3K/AKT signaling pathway were significantly downregulated in the low disulfidptosis subtype. Aberrant activation of PI3K/AKT signaling promotes tumor development, and PI3K/AKT inhibitors have shown promising results in suppressing tumors in clinical trials [48–51].

To establish a reliable model for typing HCC patients, we identified five key signature genes (*SLC7A11, SLC2A1, ADAM9, ITGAV,* and *PFKP)* that distinguished between the C1 and C2 subgroups. The constructed model based on these genes exhibited high accuracy, with an AUC value of 0.951 (95%CI 0.923–0.972) in the ROC analysis. This finding confirms the successful differentiation of HCC patients into two disulfidptosis subtypes, with the low disulfidptosis subtype associated with better prognosis.

To further explore the link between disulfrgs and immune effects, we constructed a WGCNA network integrating the expression matrix of 1006 DEGs and immune infiltration levels obtained using CIBERSORT. The BLUE module, characterized by infiltration levels of M1 and M2 macrophages, was selected from the network. Intersection analysis between the 744 genes in this module and the 588 protein-coding genes in the 619 DEPGs resulted in 503Siggs. To evaluate the disulfidptosis status of each patient, we identified five disulfidptosis-related genes (*NEIL3, MMP1, STC2, ADH4,* and *CFHR3*) from different disulfidptosis subtypes. Comparison of disulfS among patients in different disulfrg revealed significantly lower disulfS in groups C2 compared to groups C1, consistent with the previous findings of better prognosis in groups C2.

We conducted comprehensive validation of the accuracy and validity of disulfS as an independent predictor for HCC patients, demonstrating its precise and effective prediction of OS and PFS in HCC patients. The internal and external cohorts showed good agreement, further solidifying the significance of our disulfS construct. Moreover, we observed a strong association between high disulfS groups and advanced tumor grade and stage, highlighting the important implications of our findings.

We further investigated the role of high and low-risk disulfS groups in assessing MSI, mRNAsi, TIME, and GMF in HCC patients. Notably, the disulfS model exhibited a significant positive correlation with the expression levels of mismatch repair genes (*MLH1, MSH2, MSH6,* and *PMS2*). The clinical value of MSI in guiding diagnosis and treatment has been established in various tumors, including colorectal cancer [52], gastric cancer [53], and endometrial cancer [54]. High MSI often indicates poor immunotherapeutic outcomes and prognosis. Furthermore, we found a significant positive correlation between the disulfS model and mRNAsi, a measure closely related to tumor dedifferentiation. A higher mRNAsi score signifies increased tumor dedifferentiation and suggests a poorer prognosis [24]. Surprisingly, we also discovered a higher frequency of TP53 mutations, a common driver gene in HCC, among the high disulfS group. TP53 mutations are associated with worse clinical stage and prognosis in patients with HCC, particularly in Western countries [55].

Immunotherapies targeting immune checkpoints such as PD-1/PD-L1 and CTLA-4 have shown positive responses in HCC patients [56, 57]. However, the limited response rate can primarily be attributed to the constraints of tumor immune status [58]. To uncover the practical implications of disulfS in guiding clinical treatment, we analyzed IPS and immune exclusion scores of two immune checkpoint inhibitors from the TCIA database. Remarkably, the low disulfS group exhibited higher IPS and lower exclusion scores. IPS and exclusion scores serve as immune reference indicators for assessing the extent of checkpoint inhibitor benefit [59]. Additionally, we evaluated the predictive value of disulfS for immunotherapy in a PD-1-treated melanoma cohort, demonstrating its efficacy in predicting patient OS. Notably, the low disulfS group exhibited better response rates. Furthermore, our analysis of sorafenib drug sensitivity revealed an intriguing phenomenon: patients in the low disulfS group showed an antagonistic trend between the effects of sorafenib-targeted therapy and immunotherapy. This observation supports a preference for immunotherapy over sorafenib treatment in advanced HCC patients, aligning with the results of a phase III clinical trial comparing Atezolizumab plus bevacizumab to sorafenib [60].

Next, we demonstrated the occurrence of disulfidptosis in HCC cells and identified SLC7A11 and SLC2A1 as key genes through cell proliferation assays and phalloidin staining. Furthermore, our co-culture modeling indicated a potential association between disulfidptosis and immunotherapeutic response in HCC. Lastly, we validated the clinical application value of the constructed disulfS signature in our own cohort. Although several studies have attempted to establish a prognostic signature for disulfide death-related genes in HCC [16–19, 61, 62], they have primarily focused on bioinformatics analysis without conducting further molecular biology experiments to confirm the existence of disulfide death in hepatocellular carcinoma. Additionally, some of these studies constructed prognostic models with inadequate AUC values for ROC curves in validation cohorts, failing to validate the predictive effects of these models in real-world settings. To overcome these limitations, our study sought to address these gaps by carrying out cell biology experiments and collecting clinical samples from hospitals. This approach allowed us to explore the phenomenon of disulfide death in HCC more comprehensively and provided a solid foundation for our findings. In summary, our study identified two key genes, SLC7A11 and SLC2A1, crucial for the molecular typing of disulfidptosis in HCC patients. These genes exhibited excellent predictive power for patient survival. SLC7A11 serves as an important importer of cysteine for glutathione biosynthesis and antioxidant defense, and its overexpression is observed in various human cancers [63]. Increased SLC7A11 expression promotes tumor growth by suppressing ferroptosis levels [64]. Moreover, SLC7A11 overexpression synergizes with ferroptosis inducers to enhance sensitivity to PARP inhibitors in BRCA-positive ovarian cancer patients [65]. The SLC7A11-associated high-rate cysteine metabolism in tumor cells relies on the pentose phosphate pathway to generate substantial amounts of NAPDH, establishing a link to metabolic vulnerabilities that could guide therapies targeting cancers with high SLC7A11 expression [14]. On the other hand, SLC2A1 promotes immune evasion and liver metastasis in colon cancer by inducing regulatory T cells [66]. Deletion of SLC2A1 in tumor-associated neutrophils hampers lung tumor growth and enhances radiotherapy efficacy [67]. CDK6-mediated transcriptional downregulation of SLC2A1 induces autophagy in HCC cells through the AMPK-ULK1 pathway [68].

However, our study has certain limitations. Firstly, further validation of the accuracy and efficacy of disulfS in large multicenter prospective cohorts is necessary. Secondly, ongoing exploration of the specific mechanisms of action of SLC7A11 and SLC2A1 through basic experiments is a direction for future research efforts.

In conclusion, our study introduces a well-defined molecular typing of HCC patients based on disulfrg, integrating different typing methods with immune infiltration. We quantitatively constructed disulfS as a novel prognostic and therapeutic biomarker, accurately predicting prognosis and immunotherapy response in tumor patients. Furthermore, we identified key prognostic genes associated with HCC development and validated their expression in HCC, providing new insights into prognosis and treatment strategies. The disulfS scoring method can aid clinicians in developing accurate and personalized treatment plans, potentially improving patient outcomes and tailoring individualized therapies.

### Supplementary Information

Supplementary Material 1. Fig. 1: Expression and genetic alteration of disulfrgs in TCGA-LIHC. (A) Expression of 31 disulfrgs in HCC and normal tissues. (B) Mutation frequency and classification of disulfrgs in the TCGA cohort of 371 HCC patients. (C) Copy number variation of 31 disulfrgs. Distribution of 31 disulfrgs on chromosomes. *p < 0.001; ns, not statistically different.Supplementary Fig. 2: Prognosis significance of disulfrgs in HCC patients.

Supplementary Material 2. Fig. 2: Prognosis significance of disulfrgs in HCC patients. (A) Univariate Cox forest plot of 31 disulfrgs of OS in HCC patients. (B) KM survival analysis curves of 9 key significant prognostic genes.

Supplementary Material 3. Fig. 3: The GSVA heatmap showed differences of pathways between the two disulfidptosis subtypes from three genesets: (A) "c2.cp.kegg.v7.5.1.symbols.gmt". (B) "c2.cp.reactome.v7.5.1.symbols.gmt". (C) "h.all.v7.5.1.symbols.gmt".

Supplementary Material 4. Fig. 4: Enrichment analysis of DEGs between the two disulfidptosis subtypes. (A) Volcano plot of DEGs between two subtypes. (B) Circle diagram of KEGG enrichment analysis of DEGs between two subtypes. (C) Bubble diagram of GO enrichment analysis of DEGs between two subtypes. (D) Bubble diagram of KEGG enrichment analysis of DEGs between two subtypes.

Supplementary Material 5. Fig. 5: RF and ANN models to classify HCC patients into different disulfidptosis subtypes. (A) RF model to identify the disulfidptosis characteristic genes. (B) The top 30 important genes in RF model from 1006 DEGs. (C) Construction of ANN model for subtypes classification. (D) ROC curve of the ANN model.

Supplementary Material 6. Fig. 6: Prognostic risk prediction of HCC patients in TCGA by the disulfS prediction model. (A) Heat map of differential expression of 5 DPRGs and risk curves for high and low disulfS groups in the TCGA-train cohort. (B) Heat map and risk curves of differential expression of 5 DPRGs in the high and low disulfS groups in the TCGA-test cohort. (C) Heat map and risk curves of differential expression of 5 DPRGs in the high and low disulfS groups in the TCGA-all cohort. (D) Univariate Cox analysis forest plot of disulfS and common clinical characteristics. (E) Multivariate Cox analysis forest plot of disulfS and common clinical characteristics.

Supplementary Material 7. Fig. 7: Grade- and stage-related clinical subgroup analysis based on disulfS. (A, B) Proportional distribution of disulfS among different grades. (C, D) Proportional distribution of disulfS among different stages. (E, F) DisulfS-related KM survival curves for different grade subgroups. (G, H) DisulfS-related KM survival curves for different stage subgroups.

Supplementary Material 8. Table 1: Clinical characteristics of XY3-HCC-Cohort.

Supplementary Material 9. Table 2: Baseline Data Sheet for the Cohort of TCGA-LIHC.

Supplementary Material 10. Table 3: Results of univariate Cox regression analysis of the 5 model genes.

## Data Availability

All the original data and code in this article have been uploaded to NutCloud and can be accessed through the following link. https://www.jianguoyun.com/p/DUIr744QtcP1CxjemMcFIAA

## Abbreviations

HCC: Hepatocellular carcinoma
NMF: Non-negative matrix factorization
DisulfS: Disulfidptosis scoring system
OS: Overall survival
PFS: Progression-free survival
TME: Tumor microenvironment
ICIs: Immune checkpoint inhibitors
Disulfrg: Disulfidptosis-related genes
MSI: Microsatellite instability
IPS: Immunophenoscore
TMB: Tumor mutation load
CNV: Copy number variation
t-SNE: T-distributed stochastic neighbor embedding
KM: Kaplan–Meier
GSVA: Gene set variation analysis
DEGs: Differential genes
LogFC: LogFoldChange
GO: Gene ontology
KEGG: Kyoto encyclopedia of genes and genomes
BP: Biological processes
CC: Cellular components
MF: Molecular functions
ANN: Artificial neural network
ROC: Receiver operating characterastic
AUC: Area under the curve
WGCNA: Weighted gene co-expression network analysis
TOM: Topological overlap matrix
Siggs: Significant genes
LASSO: Least absolute shrinkage and selection operator
DPRGs: Disulfidptosis potentially related genes
TMB: Tumor mutation burden
MMR: Mismatch repair mechanism
TIME: Tumor immune microenvironment
IC50: Half-inhibitory concentration
GDSC: Genomics of drug sensitivity in cancer
Nec-1: Necrostatin-1
Nec-2: Necrostatin-2
CQ: Chloroquine
DFOM: Deferoxamine mesylate
IOD: Integrated optical density
ELISA: Enzyme-linked immunosorbent assay
AFP: Alpha-fetoprotein
ssGSEA: Single-sample Gene Set Enrichment Analysis
Tregs: Regulatory T cells
Tfh: T follicular helper
MDSC: Myeloid-derived suppressor cells
mRNAsi: Tumor stemness index
GMF: Gene mutation frequency
