# Supplementary figures and images for: Identifying disulfidptosis subtypes in hepatocellular carcinoma through machine learning and preliminary exploration of its connection with immunotherapy
Source: Cancer Cell Int. 2024 Jun 3;24:194. doi: 10.1186/s12935-024-03387-1 (PMC11149214; doi:10.1186/s12935-024-03387-1)

A

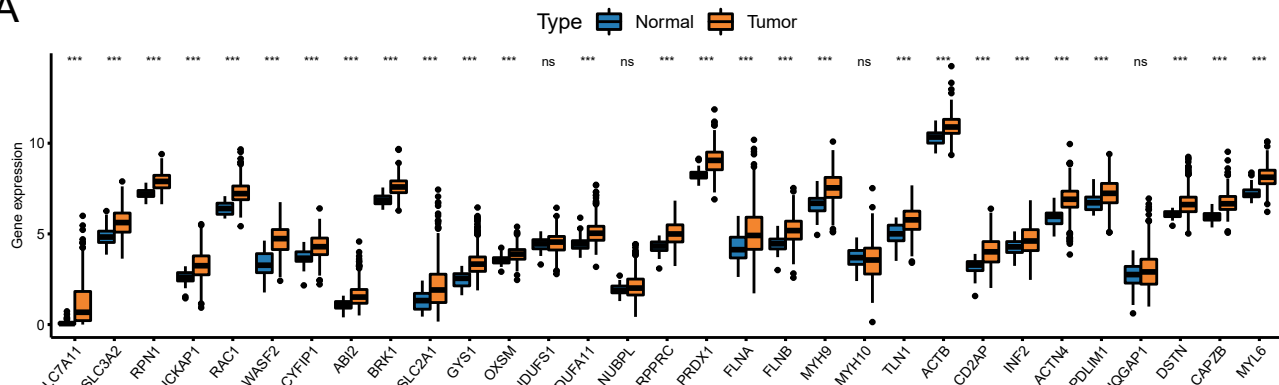

B

Altered in 54 (14.56%) of 371 samples.

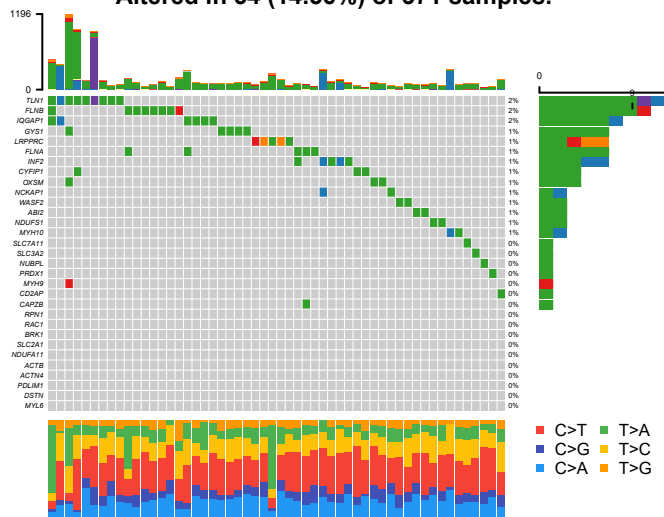

C

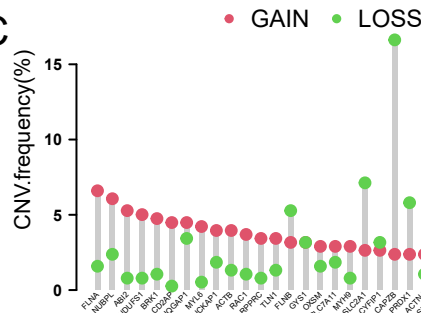

D

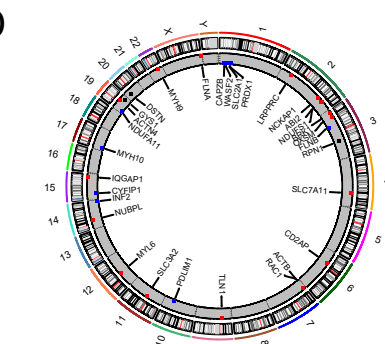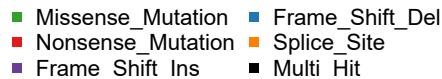

Supplement: Supplementary file 1 — Supplementary Material 1. Fig. 1: Expression and genetic alteration of disulfrgs in TCGA-LIHC. (A) Expression of 31 disulfrgs in HCC and normal tissues. (B) Mutation frequency and classification of disulfrgs in the TCGA cohort of 371 HCC patients. (C) Copy number variation of 31 disulfrgs. Distribution of 31 disulfrgs on chromosomes. *p < 0.001; ns, not statistically different.Supplementary Fig. 2: Prognosis significance of disulfrgs in HCC patients. [file 12935_2024_3387_MOESM1_ESM.pdf]

A

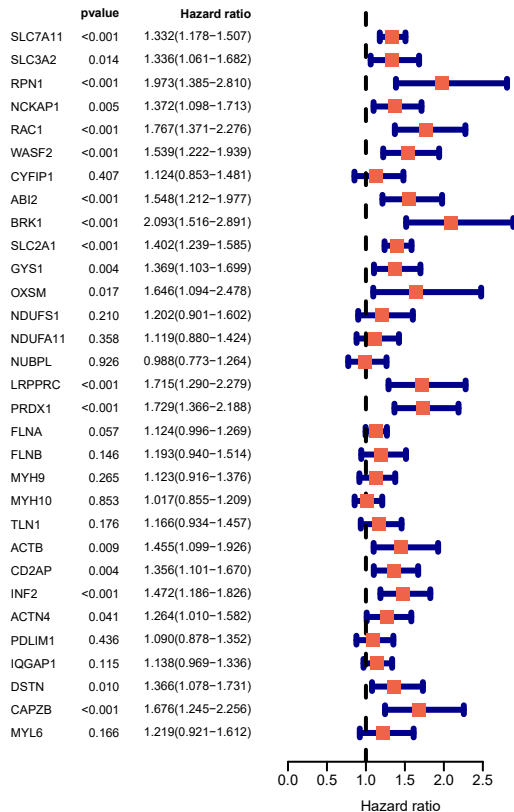

B

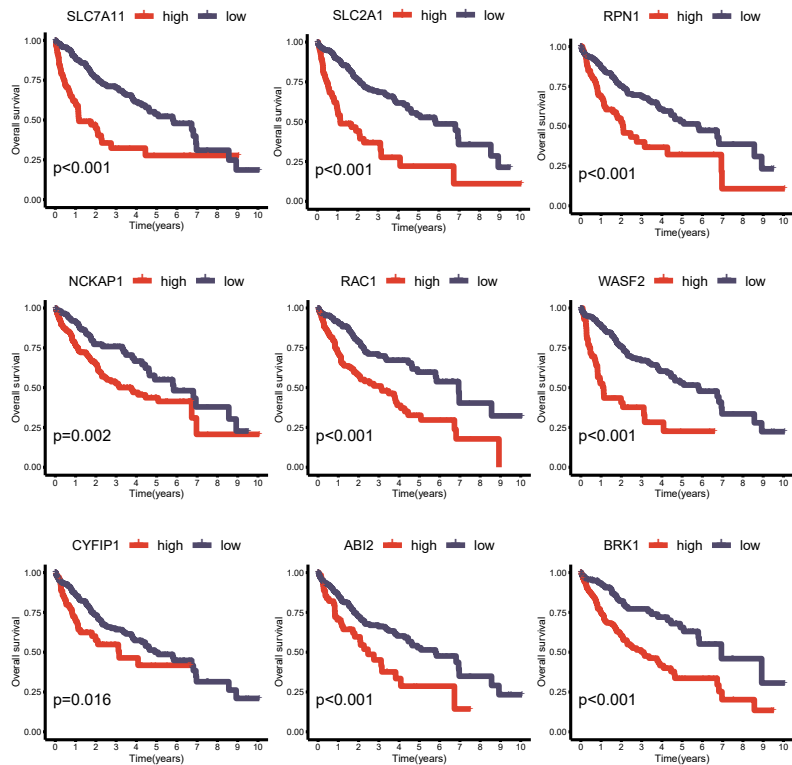

Supplement: Supplementary file 2 — Supplementary Material 2. Fig. 2: Prognosis significance of disulfrgs in HCC patients. (A) Univariate Cox forest plot of 31 disulfrgs of OS in HCC patients. (B) KM survival analysis curves of 9 key significant prognostic genes. [file 12935_2024_3387_MOESM2_ESM.pdf]

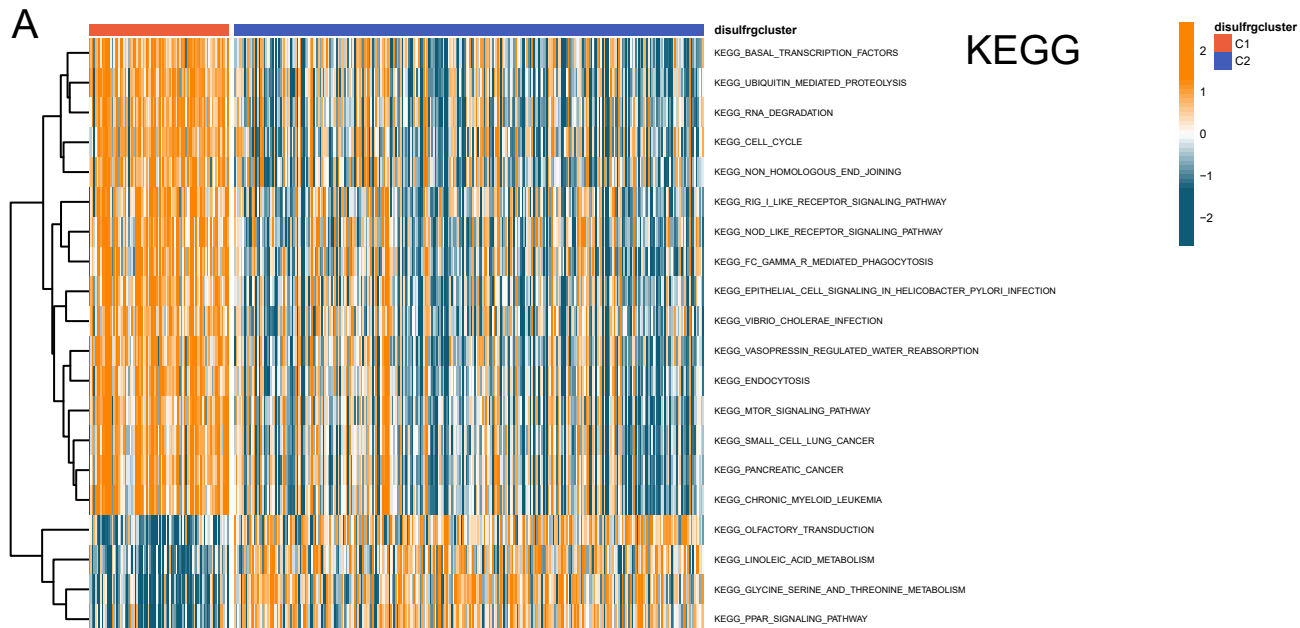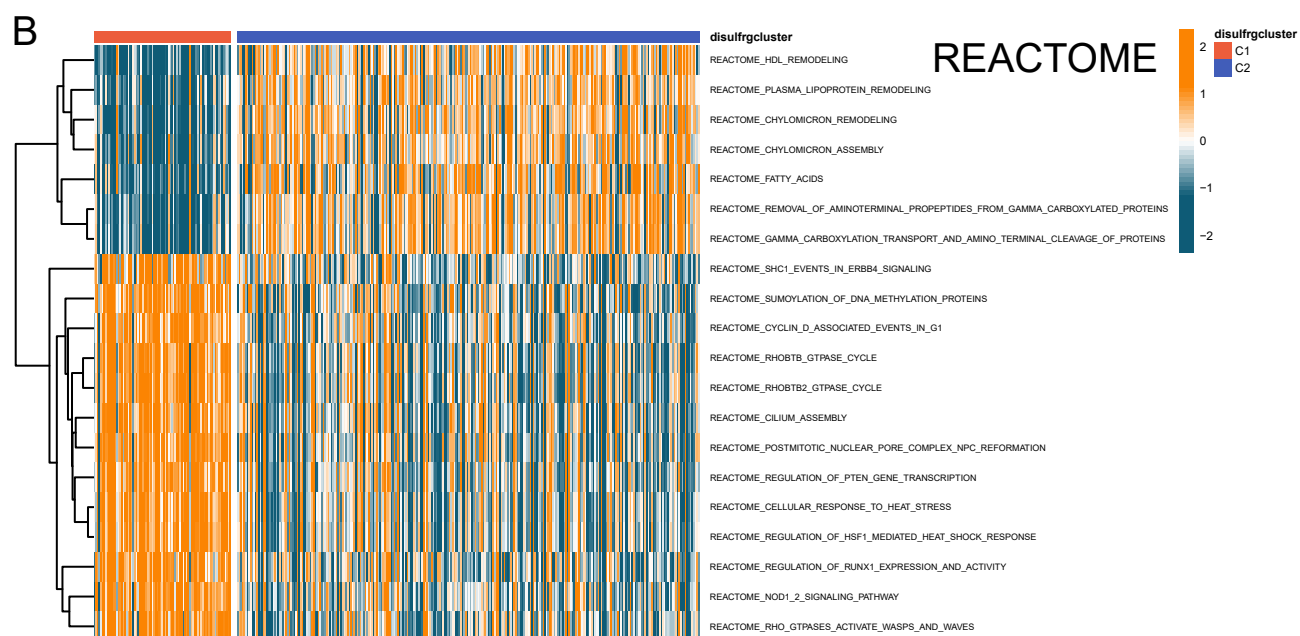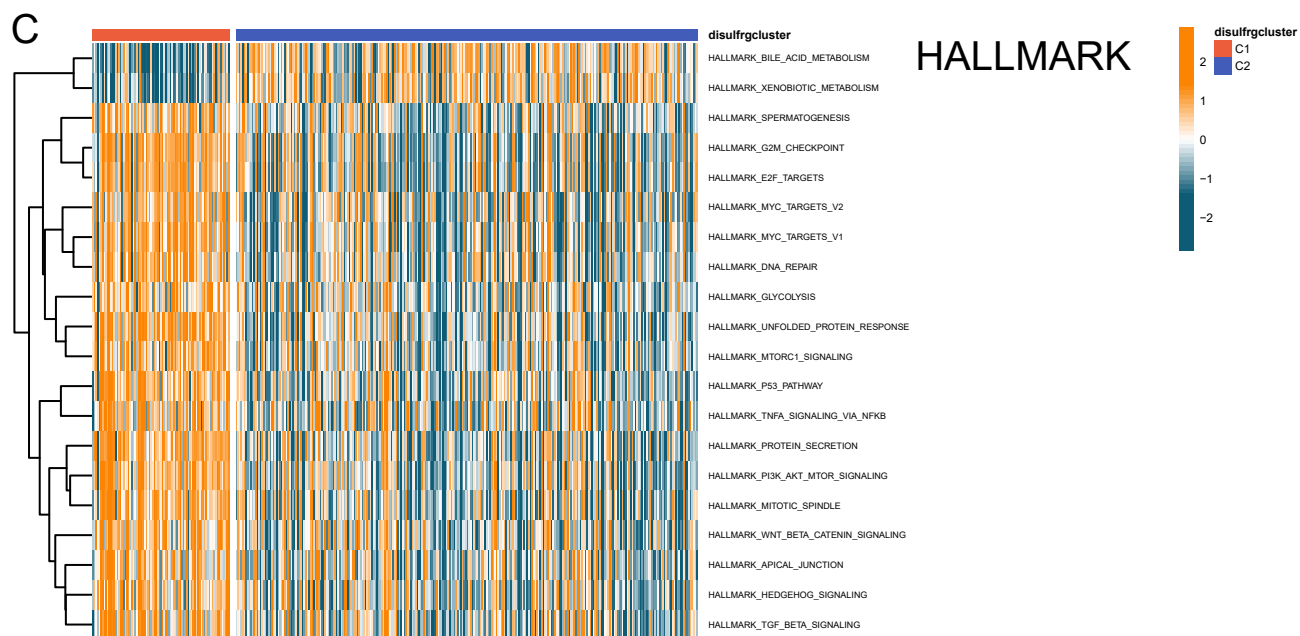

Supplement: Supplementary file 3 — Supplementary Material 3. Fig. 3: The GSVA heatmap showed differences of pathways between the two disulfidptosis subtypes from three genesets: (A) "c2.cp.kegg.v7.5.1.symbols.gmt". (B) "c2.cp.reactome.v7.5.1.symbols.gmt". (C) "h.all.v7.5.1.symbols.gmt". [file 12935_2024_3387_MOESM3_ESM.pdf]

A

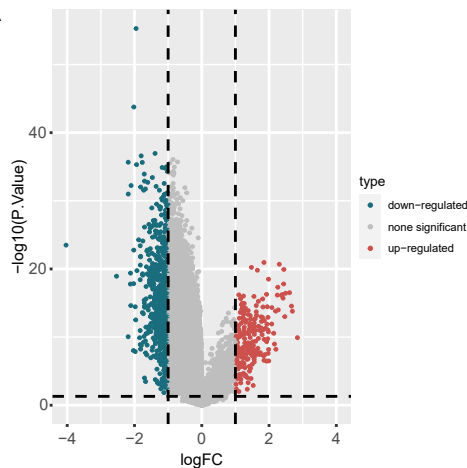

B

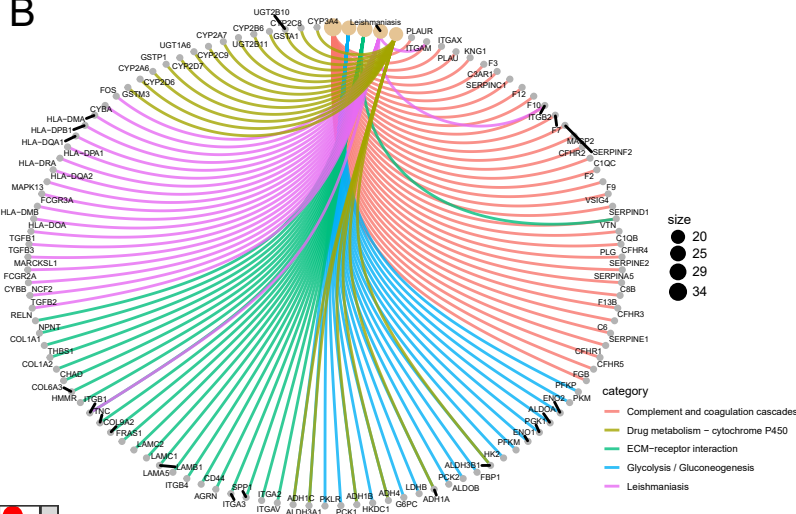

C

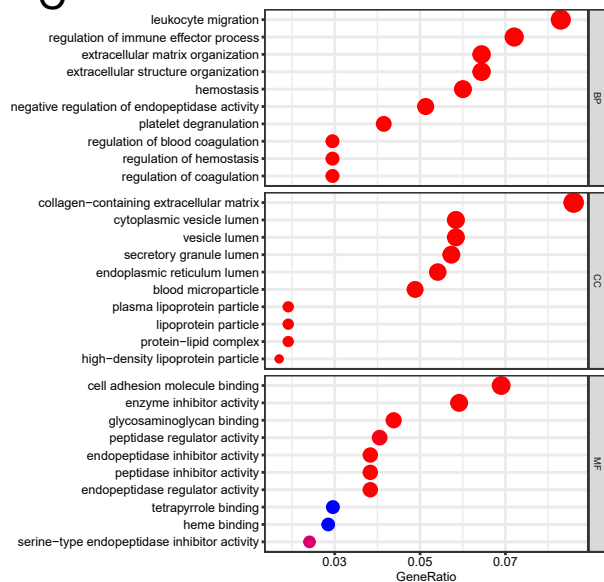

D

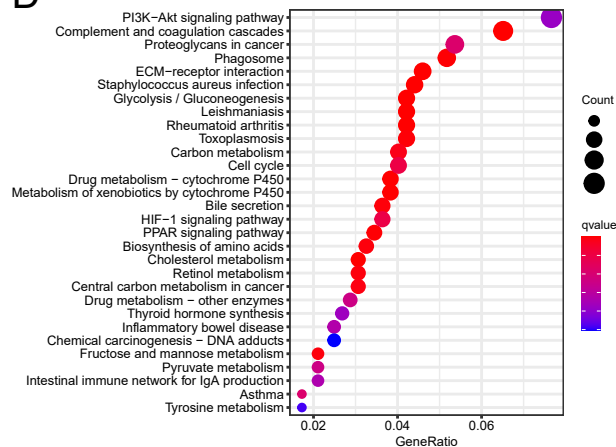

Supplement: Supplementary file 4 — Supplementary Material 4. Fig. 4: Enrichment analysis of DEGs between the two disulfidptosis subtypes. (A) Volcano plot of DEGs between two subtypes. (B) Circle diagram of KEGG enrichment analysis of DEGs between two subtypes. (C) Bubble diagram of GO enrichment analysis of DEGs between two subtypes. (D) Bubble diagram of KEGG enrichment analysis of DEGs between two subtypes. [file 12935_2024_3387_MOESM4_ESM.pdf]

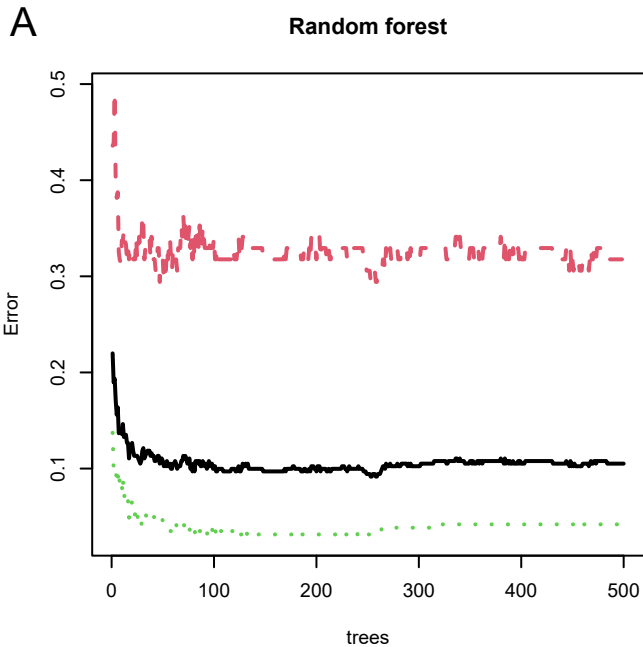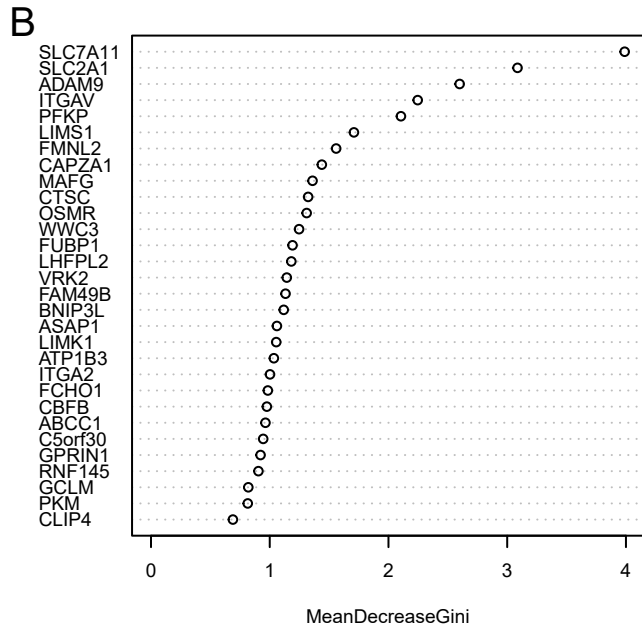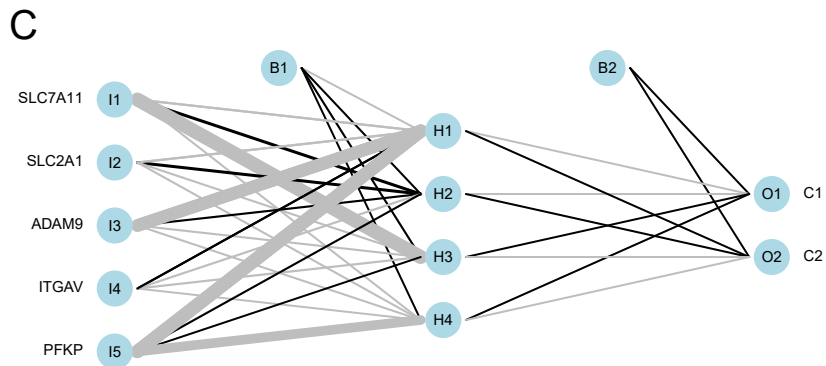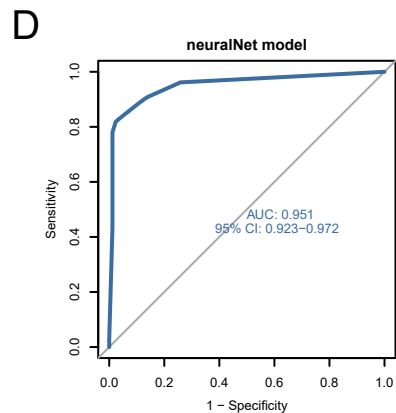

Supplement: Supplementary file 5 — Supplementary Material 5. Fig. 5: RF and ANN models to classify HCC patients into different disulfidptosis subtypes. (A) RF model to identify the disulfidptosis characteristic genes. (B) The top 30 important genes in RF model from 1006 DEGs. (C) Construction of ANN model for subtypes classification. (D) ROC curve of the ANN model. [file 12935_2024_3387_MOESM5_ESM.pdf]

**A**

Train TCGA cohort

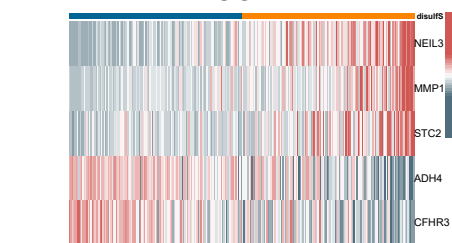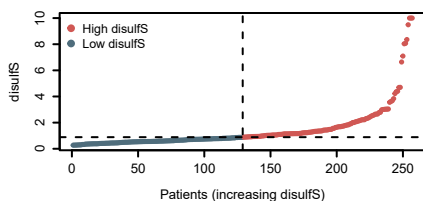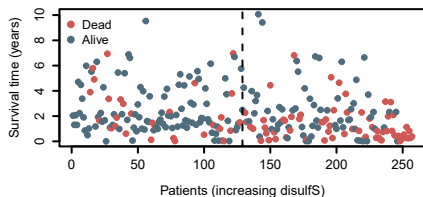**B**

Test TCGA cohort

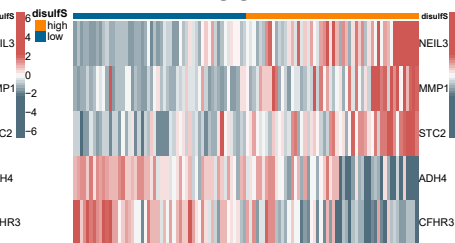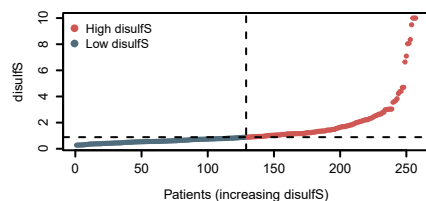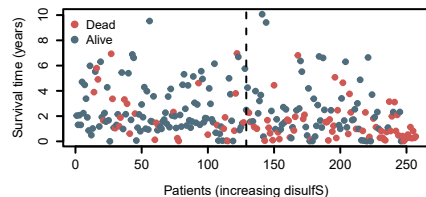**C**

All TCGA cohort

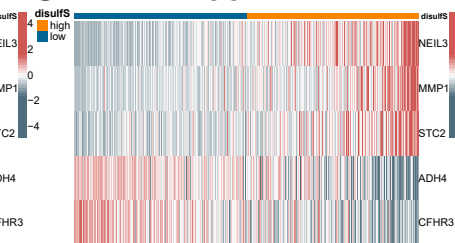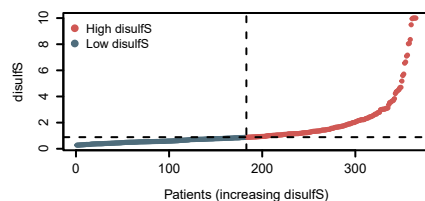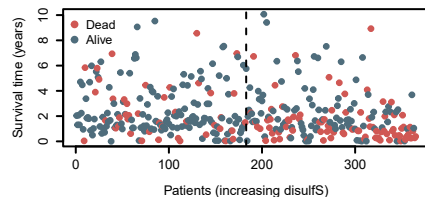**D**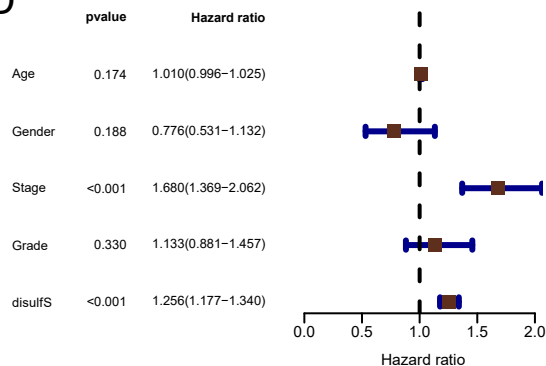**E**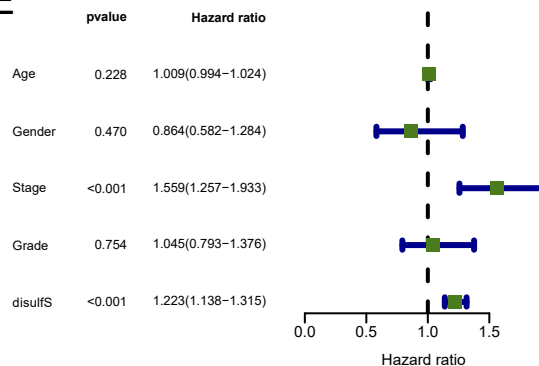

Supplement: Supplementary file 6 — Supplementary Material 6. Fig. 6: Prognostic risk prediction of HCC patients in TCGA by the disulfS prediction model. (A) Heat map of differential expression of 5 DPRGs and risk curves for high and low disulfS groups in the TCGA-train cohort. (B) Heat map and risk curves of differential expression of 5 DPRGs in the high and low disulfS groups in the TCGA-test cohort. (C) Heat map and risk curves of differential expression of 5 DPRGs in the high and low disulfS groups in the TCGA-all cohort. (D) Univariate Cox analysis forest plot of disulfS and common clinical characteristics. (E) Multivariate Cox analysis forest plot of disulfS and common clinical characteristics. [file 12935_2024_3387_MOESM6_ESM.pdf]

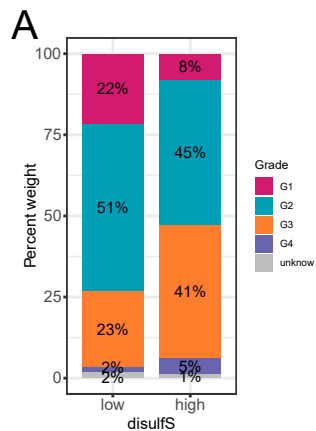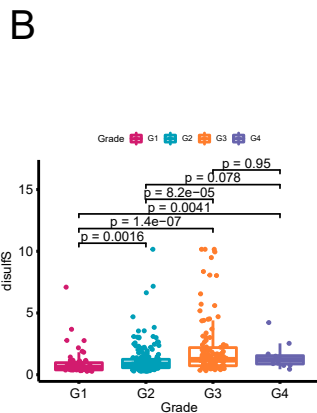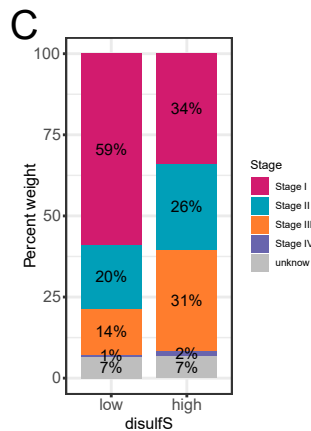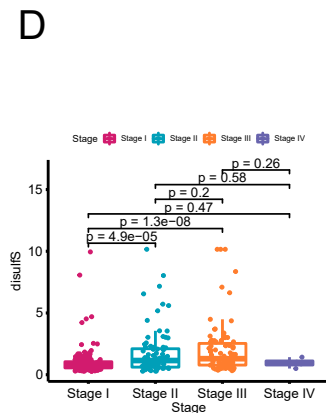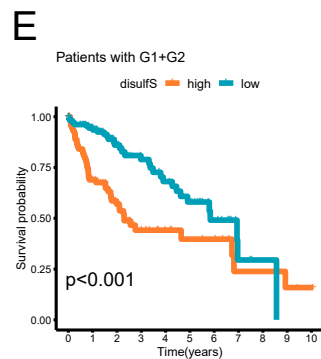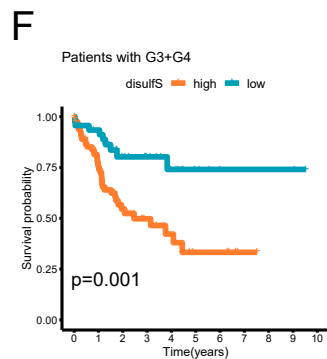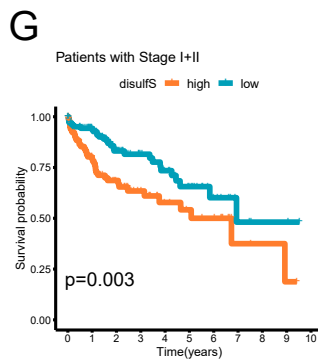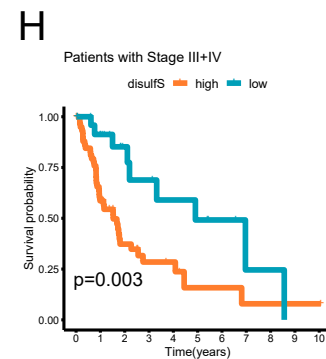

Supplement: Supplementary file 7 — Supplementary Material 7. Fig. 7: Grade- and stage-related clinical subgroup analysis based on disulfS. (A, B) Proportional distribution of disulfS among different grades. (C, D) Proportional distribution of disulfS among different stages. (E, F) DisulfS-related KM survival curves for different grade subgroups. (G, H) DisulfS-related KM survival curves for different stage subgroups. [file 12935_2024_3387_MOESM7_ESM.pdf]
